# Direct measurement of RNA Polymerase II hypertranscription in cancer FFPE samples

**DOI:** 10.1101/2024.02.28.582647

**Published:** 2024-03-16

**Authors:** Steven Henikoff, Jorja G. Henikoff, Ronald M. Paranal, Jacob E. Greene, Ye Zheng, Zachary R. Russell, Frank Szulzewsky, Sita Kugel, Eric C. Holland, Kami Ahmad

**Affiliations:** 1 Basic Science Division, Fred Hutchinson Cancer Center, Seattle, WA, USA; 2 Howard Hughes Medical Institute, Chevy Chase, MD, USA; 3 Human Biology Division, Fred Hutchinson Cancer Center, Seattle, WA, USA; 4 Molecular Medicine and Mechanisms of Disease (M3D) PhD Program, University of Washington, Seattle, WA, USA; 5 Vaccine and Infectious Disease Division, Fred Hutchinson Cancer Center, Seattle, WA, USA

## Abstract

Hypertranscription is widespread in aggressive human cancers. However detection relies on mRNAs, which are heavily processed and have variable half-lives, and on accurate cell number estimations. Previously we introduced FFPE-CUTAC, a genome-wide method for mapping RNA Polymerase II in formalin-fixed paraffin-embedded (FFPE) sections. Here we apply FFPE-CUTAC on slides and curls to demonstrate hypertranscription at regulatory elements and replication-coupled histone genes. We find that hypertranscription differs between transgene-driven mouse gliomas and scales with enhanced proliferation and reduced mitochondrial DNA. We also apply FFPE-CUTAC to identify tumor-specific patterns in assorted human tumor-normal pairs. We analyze the top-ranked 100 annotated regulatory elements that are hypertranscribed in most of the tumors and identify multiple loci around ERBB2 on Chromosome 17q12–21 in the breast and colon cancer samples, mapping likely HER2 amplifications punctuated by selective sweeps. Our results demonstrate that FFPE-CUTAC measurement of hypertranscription provides an affordable and sensitive genome-wide strategy for cancer diagnosis.

## Introduction

Global increase in nascent transcription, referred to as hypertranscription, is a general feature of cells undergoing proliferation during development (1) and has been extensively documented in cancer (2, 3). For example, high-level expression of the *cMyc* oncogene has been hypothesized to act as a transcriptional amplifier (4–6), globally increasing the frequency of transcriptional bursting at promoters of active genes within mammalian genomes (7). Global hypertranscription has also been observed more generally in aggressive human cancers under control of a wide variety of transcription factors (TFs) in addition to *cMyc* (2). Hypertranscription in cancer has been attributed to widespread loss of transcriptional repression (2), which is thought to result from an inability of topoisomerase I to prevent transcription overdrive (8). Many of these studies of genome-wide hypertranscription have relied on measurements of abundant stable mRNAs, but scaling the level of any particular mRNA to the level of its template DNA is challenging. Most hypertranscription estimates have been based on calibrating RNA-seq data using spike-ins (6), although more recently estimates have been based on polymorphisms in regions of cancer-specific loss of heterozygosity (2), on estimates of DNA abundance and ploidy in matched RNA-seq and DNA-seq samples (3) or on single-cell mRNA abundances (9). However, these estimates based on RNA-seq are complicated by variability in mRNA lifetimes and are relatively insensitive to low-level transcripts such as those for transcription factors that drive development and are misregulated in cancer. Additional challenges arise in estimating hypertranscription routinely from patient samples, the vast majority of which are fixed in formalin and embedded in paraffin, a procedure that has been the standard for well over a century (10). The days-long fixation in formalin (~4% formaldehyde) results in cross-linking and adduct formation on both RNA and DNA, which makes extraction and sequencing difficult. Although commercial kits have been developed that allow the severely damaged RNA and DNA to be efficiently extracted and sequenced, the detection of hypertranscription in FFPEs in RNA and DNA sequencing data has not been performed using these methods.

Previously, we showed that formalin treatment, rather than being a severe impediment to transcriptional profiling, could be used to our advantage by profiling the RNA Polymerase II (RNAPII) transcriptional machinery, providing a direct genome-wide map of transcription on the DNA itself (11). Specifically, we had modified our antibody-directed Cleavage Under Targets and Tagmentation (CUT&Tag) *in situ* chromatin profiling method such that for promoter and enhancer epitopes, tethered Tn5 transposase would integrate DNA sequencing adapters into nearby open chromatin regions under low-salt conditions (12). Our CUTAC (Cleavage Under Targeted Accessible Chromatin) method mapped open chromatin with especially high resolution and signal-to-noise, with best results obtained using antibodies to RNA Polymerase II (RNAPII) serine phosphate epitopes (13, 14). Serine-5 phosphorylation of the C-terminal domain (CTD) of the RPB1 subunit is a mark of paused RNAPII, and comparison to published PRO-seq data for human K562 cells revealed that RNAPII-Ser5p CUTAC maps paused RNAPII at enhancers and promoters genome-wide (12). As Ser5p is abundant on the 52 tandem heptamers comprising the CTD, paused RNAPII provides a unique 364 amino acid window of lysine-free epitopes within the heavily lysine-crosslinked nucleosomal DNA of FFPEs. We took advantage of this property of CUTAC and its preference for short open chromatin regions to perform efficient profiling of regulatory elements on FFPEs (11). FFPE-CUTAC allowed for high-confidence identification of known biomarkers in mouse brain cancers, including microRNA tumor suppressor loci invisible to standard RNA-seq. FFPE-CUTAC using both RNAPII-Ser5p and histone H3K27ac antibodies on FFPEs also provided much better biomarker discrimination than RNA-seq on fresh mouse brain tumor samples.

Our FFPE-CUTAC protocol was performed either on magnetic beads or directly on slides through the tagmentation step, then tissue was scraped into PCR tubes for fragment release and amplification. Here we show that FFPE-CUTAC on slides can be used to directly map hypertranscription at regulatory elements throughout the mouse genome, revealing that the degree of hypertranscription varies between genetically identical tumors and for some is not observed at all. We also discovered that FFPE-CUTAC distinguishes tumors based on RNAPII at replication-coupled histone genes and reduced mitochondrial DNA abundance relative to matched normal tissues, likely a consequence of selection for reduced oxidative phosphorylation. In addition, we present a new FFPE-CUTAC protocol for curls and show that it provides RNAPII-Ser5p data of especially high quality. When applied to tumor and adjacent normal 5 micron ~1 cm^2^ human FFPE sections from seven anonymous individual human tumors, FFPE-CUTAC analyzed for hypertranscription identified dozens of strongly hypertranscribed loci in common among the tumors. Strikingly, in two of the seven individual tumors we observed broad increases of RNAPII within Chromosome 17q1.2–2.1, which includes the *ERBB2* (*HER2*) locus. These evident *HER2* amplifications were punctuated with broad hypertranscribed summits, some centered over tumor driver promoters, suggestive of linkage disequilibrium caused by selective sweeps during tumor evolution. The ability of FFPE-CUTAC to precisely localize patterns of regulatory element hypertranscription with sparse material, and to map megabase-sized regions of amplification punctuated by smaller regions of likely clonal selection, makes it suitable for general personalized medicine applications.

## Results

### Fold-change upregulation of RNAPII does not accurately reflect tumor cell abundance

**Figure 1 |:**
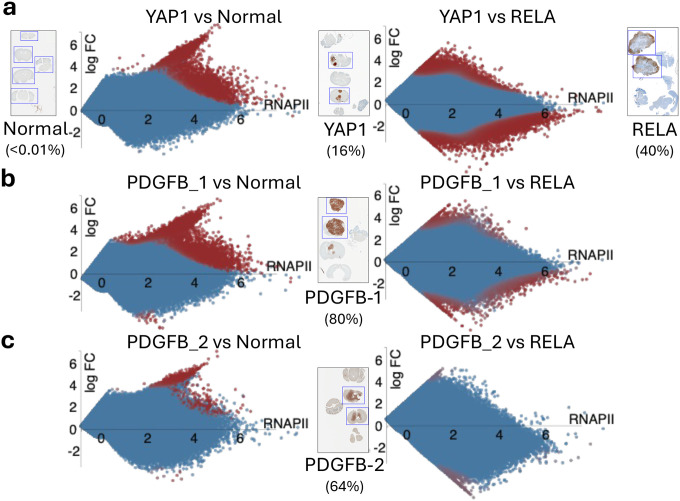
RNAPII-S5p hypertranscription in mouse brain tumors. Voom/Limma was used to construct MA plots based on individual 10 μm sections from single slides corresponding to the boxed sections on slides DAP-stained for tumor-driver transgene expression. Numbers parentheses are percentages of tumor cells based on numbers of stained and unstained cells within the boxed sections.

In our previous FFPE-CUTAC study (11), we observed that significantly upregulated cCREs were more frequent than downregulated cCREs in mouse brain tumors with different transgene drivers: a ZFTA-RELA (RELA) gene fusion overexpressing a transcription factor resulting in an ependymoma (15), a YAP1-FAM118b (YAP1) transcriptional co-activator gene fusion driving an ependymoma (16), and overexpression of the tyrosine-kinase active PDGFB ligand driving a glioma (17). Upregulation bias based on RNAPII fold-change is observed in pooled data from several on-slide experiments in which tumor-rich sections were separated from normal sections, although the bias is much greater for RELA than for PDGFB (Supplementary Figure 1). To further understand this difference and to eliminate sample-to-sample variability, we examined on-slide dissection data from single FFPE slides representing normal mouse brain, RELA and YAP1 tumors and PDGFB tumors from two genetically identical mice. Unexpectedly, the fold-change upregulation bias showed little relationship to the percentage of tumor in the sample as determined by counting cells stained for tumor transgene expression. Specifically, YAP1 tumor sections averaged 16% tumor cells (Figure 1a) and showed similar bias to the PDGFB-1 sections with 80% tumor cells (Figure 1b) and stronger upregulation bias than the PDGFB-2 sections with 64% tumor cells (Figure 1c), and all three showed weaker upregulation versus the RELA tumor sections with 40% tumor cells (Figure 1a–c, left panels). Fold-change is the ratio of tumor:normal, which does not distinguish between a weak signal increasing to moderate strength and a moderate signal increasing to high strength. We considered the possibility that the differences in RNAPII upregulation that we observed arise from incremental increases in the normal transcription levels of the targets of these different cancers.

### Global hypertranscription of cCREs scales with tumor abundance in mouse brain tumors

**Figure 2 |:**
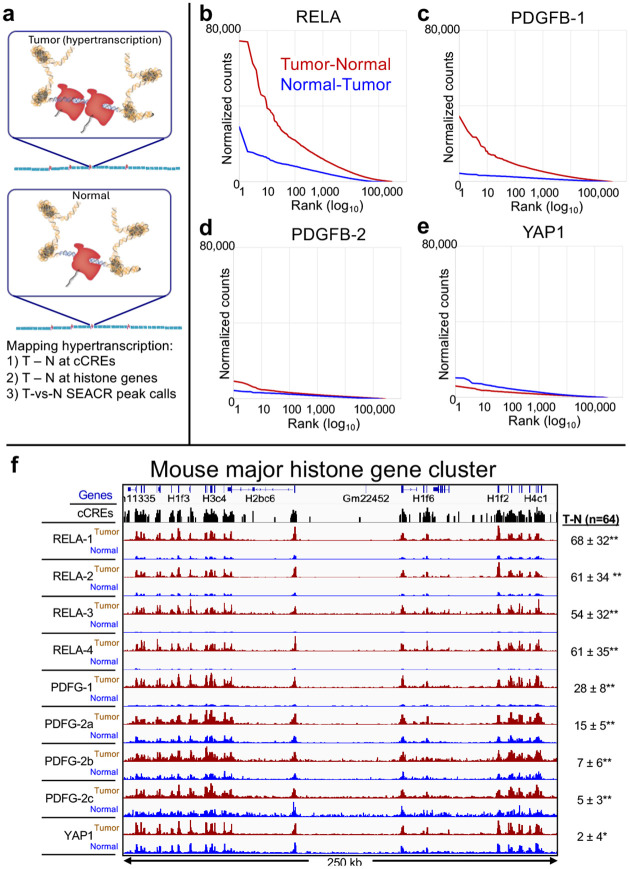
RNAPII-Ser5p FFPE-CUTAC directly maps hypertranscription across the mouse genome. **a)** Model for global hypertranscription in cancer: Paused RNAPII at active gene regulatory elements such as promoters and enhancers increases on average over the cell cycle resulting in a net gain in RNAPII occupancy across the genome. Using RNAPII FFPE-CUTAC we can map hypertranscription genome-wide using three complementary approaches: 1) Genome-scaled Tumor (T) minus Normal (N) counts at cCREs, 2) T – N at replication-coupled histone genes and 3) SEACR Tumor peak calls using Normal as the background control. **b-e**) Hypertranscription mapped over the 343,731 annotated mouse cCREs for tumor and normal sections dissected post-tagmentation from a 10 micron FFPE slice from each of the four different paraffin blocks described in Figure 1. Hypertranscription of a cCRE is defined as the excess of RNAPII-Ser5p in the indicated tumor over normal (Tumor minus Normal in normalized count units for Mm10-mapped fragments pooled from the same slide). **f**) Data from multiple technical replicate RNAPII-S5p FFPE-CUTAC samples in single experiments were pooled for each Tumor and Normal pair, where the Tumor-containing sections were dissected from the Normal sections on single slides post-tagmentation. Slides used for PDGFB-2a-c were from the same paraffin block but used in different experiments, and all others were from different paraffin blocks. Numbers at right were obtained by subtracting the sum of normalized counts in the normal sections from that in the tumor sections over all 64 annotated single-exon replication-coupled histone genes, where the Standard Deviation is shown. Paired *t*-test: * *p* < 0.001; ** *p* < 0.00001.

An alternative explanation for the differences that we see between mouse brain tumors is that they differ in the degree of global upregulation driven by the different transgenes. RELA is a transcription factor, YAP1 is a co-activator and PDGFB is a ligand for a signaling receptor, and their effects on their various downstream targets are likely to differ. Our RNAPII FFPE-CUTAC assay is well-suited to detect differences in global upregulation (Figure 2a), as it provides a sensitive DNA readout of transcription at the 343,731 mouse cCREs, unlike RNA readouts that require calibration to the DNA template. By subtracting the baseline RNAPII-Ser5p abundance in normal tissue from the abundance in tumor at each base-pair spanned by a cCRE and scaled to the mouse genome sequence (normalized counts), we can determine absolute increases in RNAPII at each cCRE. Accordingly, we rank-ordered cCREs based on Tumor minus Normal representing global upregulation, and conversely rank-ordered cCREs based on Normal minus Tumor representing global downregulation. With such a large collection of loci, our *a priori* expectation is that the rank-ordered distribution of differences between tumor and normal will be approximately the same regardless of whether the differences are based on tumor minus normal or normal minus tumor. For clarity, we plot rank-ordered differences on a log_10_ scale. Contrary to random expectation we observed striking differences in the tumor samples (Figure 2b–e). Whereas RELA showed excess RNAPII at the 100,000 most upregulated cCREs in tumors, YAP1 showed a small RNAPII deficiency for the 1,000 most upregulated cCREs. Interestingly, PDGFB tumors differed in global upregulation, strongly in PDGFB-1 and very weakly in PDGFB-2.

**Figure 3 |:**
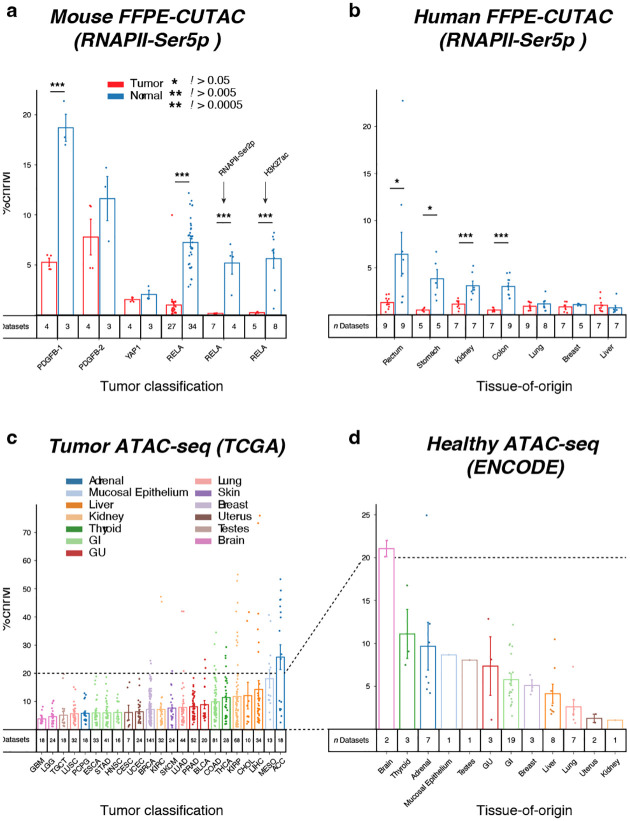
FFPE-CUTAC mitochondrial DNA signal is reduced in tumors. **a**) The percentage of normalized counts mapping to Chromosome M (ChrM = mitochondrial DNA) was calculated for FFPE-CUTAC data from four mouse brain tumor paraffin blocks driven by PDGFB, YAP1 and RELA transgenes. An RNAPII-Ser5p antibody was used for the first four comparisons, and an RNAPII-Ser2p and histone H3K27ac antibodies were used respectively for the fifth and sixth comparisons. **b**) Same as (a) for RNAPII-Ser5p FFPE-CUTAC data for the seven human Tumor/Normal pairs used in this study. **c-d**) ATAC-seq count data from TCGA (tumor) and ENCODE (normal) shows variability in ChrM percentages between tumors, consistent with our finding based on FFPE-CUTAC.

To determine whether global hypertranscription assayed by RNAPII abundance over cCREs is specific to any particular class of regulatory element(s), we divided up the data presented in Figure 3 into the five ENCODE-annotated categories: Promoters (24,114), H3K4me3-marked cCREs (10,538), Proximal Enhancers (108,474), Distal Enhancers (211,185) and CTCF cCREs (24,072). We observed that the five global RNAPII hypertranscription profiles are highly consistent with one another and with the overall cCRE profiles (Supplementary Figure 2), which suggests that RNAPII abundance differences between tumors and normal brain are independent of regulatory element function.

### Upregulation of replication-coupled histone gene transcription in mouse brain tumors

To verify that these differences in global upregulation of cCREs are related to tumor growth, we examined the profiles of the replication-coupled histone genes, which provides an independent measure of cell proliferation. In total, these small single-exon genes produce RNAPII-dependent U7-processed single-exon mRNAs during S-phase to encode for the histones that package the entire genome in nucleosomes, and so the abundance of RNAPII at these histone gene loci provides a proxy for steady-state DNA synthesis genome-wide. Of the 64 mouse replication-coupled genes, 54 are within the major histone gene cluster on Chromosome 13, and when Tumor and Normal dissection data from multiple experiments are displayed, we see differences between tumor samples consistent with our observation of global RNAPII hypertranscription differing between samples (Figure 2f). For quantitative validation, we calculated the excess of normalized counts for each experiment, with strongly significant increases for the four RELA and three PDGFB-2 biological replicates and for PDGFB-1, with a small weakly significant increase for YAP1. The consistency between our measurements of global hypertranscription over gene regulatory elements and S-phase-dependent hypertranscription over histone loci confirm that global hypertranscription is a real, but highly variable tumor-specific property of transgene-driven mouse tumors. As these exceptionally S-phase-dependent histone loci are expressed in proportion to the amount of DNA that is replicated at each cell cycle, we conclude that RNAPII-dependent hypertranscription in transgene-driven mouse cancers measures cell proliferation.

### An unsupervised method for mapping global hypertranscription

We wondered whether our observations of hypertranscription in cancer based on annotated cCREs and histone genes could be generalized using an approach that does not depend on annotations of any kind. Previously, our lab introduced SEACR (Sparse Enrichment Analysis for CUT&RUN), which was designed for application to low read-count data (18). SEACR optionally uses a background control dataset, typically for a non-specific IgG antibody. To customize SEACR for hypertranscription in cancer, we replaced the background control with the normal sample in each pair. When we merged fragment data, removed duplicates and equalized read numbers for mouse samples, SEACR called 4,226 peaks for PDGFB1, 2,382 peaks for PDGFB2 and 23,267 peaks for RELA, whereas when we ran SEACR on normal samples using tumor as background, we obtained 2, 0 and 3 peaks, respectively. These results are consistent with hypertranscription seen for these samples in Figure 2b–d. In contrast, the SEACR results for YAP1 were opposite, with 0 peaks for Tumor with Normal as background and 6702 peaks for Normal with Tumor as background, consistent with hypotranscription seen for this sample in Figure 2e.

### Mitochondrial DNA FFPE-CUTAC signal is reduced in mouse brain tumors

Mitochondrial DNA is a common contaminant of Tn5-based genomic profiling methods, because the ~16.5 kb mitochondrial DNA circles are entirely free of nucleosomes, which renders them “open” and accessible for binding of free Tn5 (19). In the case of CUT&Tag and CUTAC, the use of a 300 mM NaCl-containing buffer for Protein A-Tn5 incubation and stringent washes prevents open chromatin binding, thus minimizing mitochondrial DNA binding, which for FFPE-CUTAC is typically in the 5–10% range (11). However, in on-slide dissections, we observed a much lower level of mitochondrial DNA recovery in tumor samples than in normal samples from the same slide using RNAPII-Ser5p FFPE-CUTAC (Figure 3a). Differences in mitochondrial DNA recovery varied between mouse brain tumors, ranging from an average of 75% of normal for YAP1 down to 14% for RELA, where PDGFB-1 (28%) and PDGFB-2 (67%) were intermediate. As these differences anti-correlate with histone locus hypertranscription values (Figure 2f; R^2^ = 0.9), it seems most likely that the reductions correspond to reduced mitochondrial DNA levels in rapidly proliferating mouse cancers, which rely on glycolysis to generate ATP, and therefore would be under relaxed selection to maintain the full complement of mitochondrial DNA circles (20). We considered the alternative possibility that leakage of freely diffusible RNAPII into the mitochondrial compartment might be responsible for mitochondrial reads in FFPE-CUTAC, and an anticorrelation in cancer cells might be due to hypertranscription reducing the amount of free RNAPII in cells. Consistent with this possibility, FFPE-CUTAC using the elongating form of RNAPII (RNAPII-Ser2p) showed very strong reductions for RELA Tumor (3% of Normal). However, we also observed very strong reductions for histone FFPE-CUTAC using H3K27ac (4% of Normal), so clearly diffusion of free RNAPII cannot account for such a strong reduction in a mitochondrial FFPE-CUTAC histone modification signal in RELA tumors. Rather, it is far more likely that reduced signals reflect reduced mitochondrial DNA in these mouse brain cancers.

### Adaptation of CUTAC to FFPE curls

**Figure 4 |:**
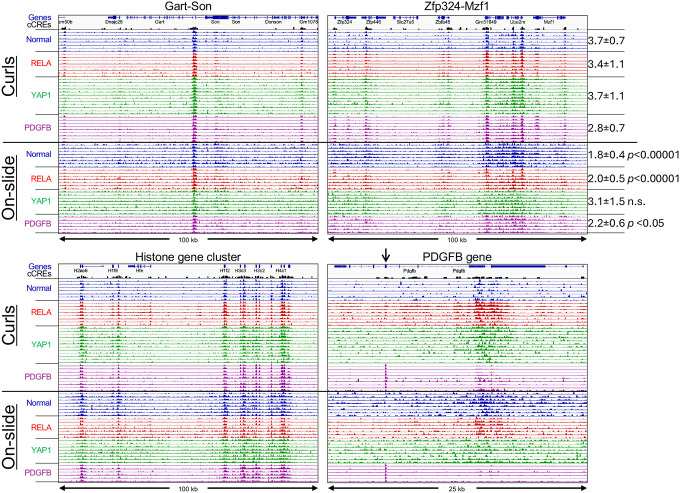
Robust RNAPII-S5p profiles using FFPE-CUTAC on curls. Tracks from representative housekeeping gene regions illustrate data quality and reproducibility. Samples from 8 curl experiments (top 36 tracks in each panel) using a variety of conditions provided high-quality data with higher signal-to-noise when compared to data produced in a single representative experiment using the on-slide protocol with slides from the same paraffin block (bottom 29 tracks). Tracks are colored according to genotype. To quantify data quality, the normalized counts in each of the 324,731 cCREs (black peaks) representing the gene regulatory 3.4% of the Mm10 genome build were summed over the mouse genome, and the degree of global enrichment relative to expectation is shown at the right. Tracks are autoscaled to maximum peak height to illustrate reproducibility despite differences in the number of mapped fragments between samples. The arrow over Exon 5 of the *Pdgfb* gene marks the coding region of the transgene that drives the tumor, providing an internal control.

Our original FFPE-CUTAC protocol was performed by xylene-based deparaffinization on slides followed by cross-linking reversal in aqueous buffer at 80–90°C, then scraped into tubes and fragmented by needle extraction (11). To immobilize the tissue fragments and to allow for stringent washes required for CUT&Tag, we added Concanavalin A-conjugated ~1.6 μm polystyrene paramagnetic beads. While the beads proved to be efficient at capturing the tissue fragments, they also captured low-level contaminants resulting in preferential amplification of (uncrosslinked) bacterial DNA that evidently contaminated the paraffin used for embedding. To overcome this problem, we tested unconjugated beads, and found that they bound fragments weakly, presumably by electrostatic bonding to the amine coating. We also tested much larger (10–40 μm) agarose beads functionalized with glutathione and found that they bound less well. In both cases, brief centrifugation in a microcentrifuge before magnetization helped to adhere tissue fragments to the side of a PCR tube. However, this protocol cannot be adapted directly to curls without removal of the paraffin. For that, we developed a protocol in which a small volume of mineral oil is added together with amine-coated 1.6 μm beads to a tube containing a curl, heated briefly to 90°C to melt the paraffin, followed by brief homogenization using a pestle motor with a disposable pestle that tightly fits the tube. Cross-linking reversal buffer (800 mM Tris-HCl pH 8.0 (21)) is added and incubation continued at 85–90°C for 1 hour or longer. After centrifugation, excess mineral oil is removed from the top, and 1–10 μm agarose-coated paramagnetic beads are added, followed by the standard CUT&Tag protocol using the CUTAC modification with low-salt tagmentation. We applied this protocol to 10 μm thick FFPE tissue section curls from paraffin blocks of normal mouse brain, YAP1 and RELA and PDGFB. We varied bead combinations, heating temperatures (80°–90°) and durations (1 hr to 14 hr) and obtained similar high-quality results regardless of the specific protocol parameters (Figure 4). To quantitatively compare signal-to-noise between protocols, we summed the total normalized counts spanned by all 343,731 mouse candidate *cis*-regulatory elements (*c*CREs) annotated by the ENCODE project (22) and spanning in total ~3.4% of the Mm10 genome build. We found that for three of the four genotypes, normalized count enrichment relative to expectation was significantly better using the curl protocol than using the on-slide protocol for samples from the same paraffin block (Figure 4, top right).

### Global hypertranscription varies between human tumors

**Figure 5 |:**
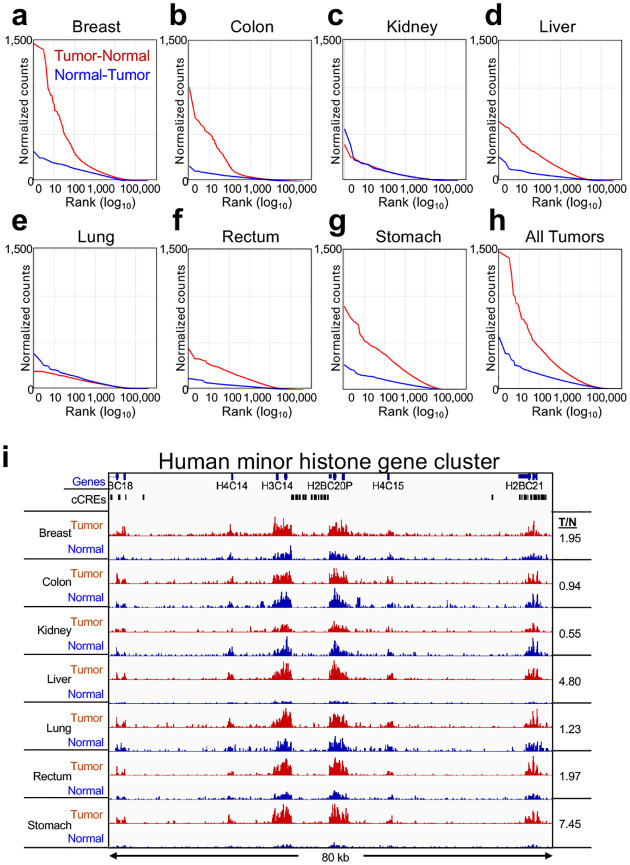
Hypertranscription in human Tumor-vs-Normal tissues: **a-h**) All fragments were pooled from four slides from the same paraffin block and the number of fragments equalized between tumor and normal for each of the seven cancers. After repeat-masking, ENCODE-annotated cCREs were rank-ordered based on average normalized counts. **i**) The minor human histone gene cluster on Chr 1 is shown, where tracks are autoscaled for each Tumor (red) and Normal (blue) pair. The liver and stomach Tumor/Normal pairs show the greatest increases in RNAPII abundance, and the kidney sample shows a conspicuous decrease, confirmed by the ratio of average Tumor/Normal counts over the entire region (numbers at right) between different hepatocarcinomas (Tumor 1: dotted lines, Tumor 2 solid lines, where tumor is red and normal is blue).

To expand on our findings of hypertranscription and mitochondrial DNA reductions based on transgene-driven mouse brain tumors to naturally occurring cancers, we obtained 5 μm FFPE sections on slides prepared from paraffin blocks of anonymous human tumor and adjacent normal pairs (Supplementary Figure 3). We performed RNAPII-Ser5p FFPE-CUTAC using the on-slide protocol, and rank-ordered each pair by Tumor minus Normal differences to test for RNAPII hypertranscription based on the 984,834 ENCODE-annotated human cCREs. To avoid possible imbalances in the comparisons between tumor and normal pairs, we removed cCREs in repeat-masked regions of the hg19 build, pooled the data from all four independent experiments and equalized the number of fragments between tumor and normal samples. We observed clear hypertranscription of the more than 10,000 top cCREs in five of the seven samples and for the composite of all samples (Figure 5a–h): In contrast, the kidney and lung tumors showed essentially no hypertranscription. To evaluate the robustness of these hypertranscription results, we plotted hypertranscription for the data from a single slide for each specimen, and despite sparse data owing to the ~1 cm^2^ size of the 5 μm sections we observed very similar results (Supplementary Figure 4a–h). We also obtained very similar results when we removed duplicates and equalized the number of fragments for each tumor-normal pair (Supplementary Figure 4i–p).

As was the case for the mouse tumors, we obtained confirmation by examining hypertranscription over the human replication-coupled histone loci, and although the data were relatively sparse, we could confirm that our breast, liver, rectum and stomach cancer samples showed prominent hypertranscription, with kidney showing hypotranscription and lung showing little if any difference in RNAPII abundance over the ~80-kb region spanning the human minor histone cluster (Figure 5i). Unexpectedly, our colon cancer sample, which showed strong global hypertranscription, was an exception, with no detectable difference between tumor and normal at the histone loci. Together, our results suggest that global hypertranscription is a common but not a universal feature of human cancers, and that FFPE-CUTAC can sensitively measure the phenomenon in small sections of the type that are commonly used by pathologists for cytological staining and analysis.

Most of the human tumor samples showed reductions in mitochondrial DNA relative to their matched normal samples, although in contrast to the anti-correlation with hypertranscription in mouse brain tumors we observed a positive correlation (R^2^ = 0.6) for the various human tumors (Figure 3b). To reconcile these differences between mouse and human, we considered the possibility that our mouse comparisons are between different brain tumors, where oxidative phosphorylation has been proposed to be required for tumor growth (23), but our human comparisons are between radically different tumors. To test this possibility, we asked whether existing human ATAC-seq data show similar variations in mitochondrial DNA recovery between different tumors as what we detected using FFPE-CUTAC. Indeed, this appears to be the case: By mining publicly available ATAC-seq data from both the TCGA and ENCODE projects, we measured differences in recovery of mitochondrial DNA similar to differences that we observed for FFPE-CUTAC (Figure 3c–d). In the case of TCGA tumor data, the percentage of mitochondrial DNA (Chromosome M) ranges from ~4% for glioblastoma, a brain cancer, to ~25% for adrenal carcinoma, whereas for ENCODE data, which is from healthy individuals, percentages range from ~1% for kidney to ~21% for brain. This 6-fold higher level of mitochondrial ATAC-seq signal in normal brain in the ENCODE data over that of glioblastoma in the TCGA data is consistent with decreased mitochondrial DNA abundance in most human and mouse tumors in our FFPE-CUTAC data.

### Most hypertranscribed regulatory elements are shared between diverse human cancers

For SEACR-based analysis of hypertranscription in cancer, we replaced the background control with the Normal sample in each pair, merged fragment data, removed duplicates and equalized read numbers for our seven human Tumor/Normal pairs. SEACR reported a median of 4483 peaks, and when Tumor and Normal were exchanged, a median of only 15 peaks was reported, indicating that hypertranscription typically dominates over hypotranscription in human tumors. Therefore, we can use SEACR Tumor/Normal peaks as an unbiased method for discovering specific hypertranscribed loci in human cancer samples.

**Figure 6 |:**
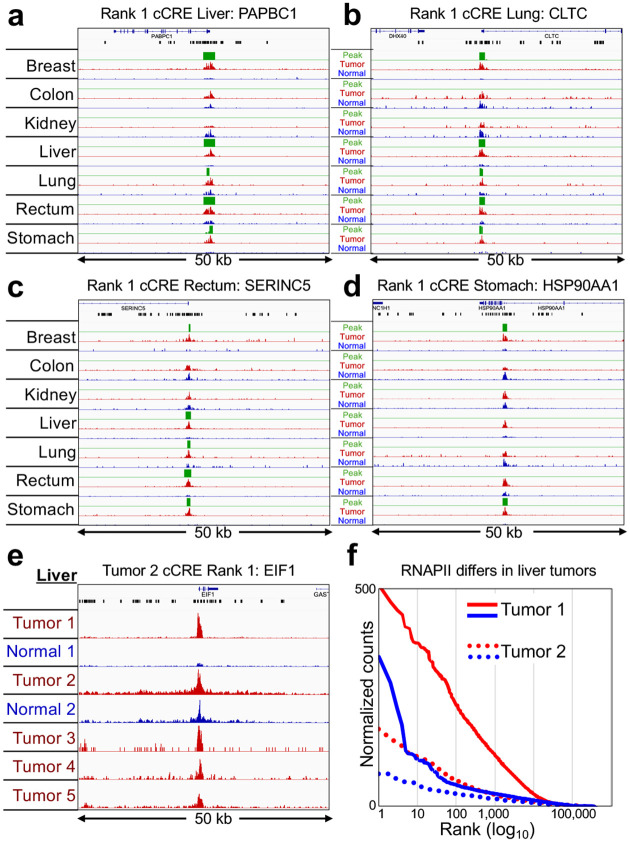
Top-ranked human cCREs based on hypertranscription correspond to SEACR Tumor-vs-Normal RNAPII-Ser5p peaks. **a-d**) For each of the indicated tumors, tracks are shown for 50-kb regions around the #1-ranked cCRE based on Tumor (red) and Normal (blue) counts, Most Tumor peaks are confirmed by SEACR (green) using Normal as the negative control for each pair. Gene annotations and cCREs (black rectangles are shown at top. **e**) Same as (a), except for top-ranked cCREs based on Liver Tumor 2. Tumor/Normal tracks and Tumors 3–5 are group-autoscaled. **f)** Levels of hypertranscription differ between different hepatocarcinomas (Tumor 1: solid lines, Tumor 2 dotted lines, where tumor is red and normal is blue).

We first asked whether SEACR Tumor/Normal peak calls corresponded to the 100 top-ranked cCREs in the overall list representing all seven tumors. Remarkably, all 100 cCREs at least partially overlapped at least one SEACR Tumor/Normal peak call, and in addition, the large majority of the 100 top-ranked cCREs intersected with overlapping SEACR peak calls from multiple Tumor/Normal pairs (Supplementary Table 1). For example, each of the #1-ranked cCREs in the liver, lung and rectum tumor samples respectively intersected gene promoters of PABPC1, CLTC and SERINC5 genes and also overlapped SEACR peak calls in 5 of the 7 tumors (breast, liver, lung, rectum and stomach, Figure 6a–c). Additionally, the #1-ranked cCRE in the stomach sample intersected an intergenic enhancer in the HSP90AA1 gene and also overlapped SEACR peak calls in both breast and stomach (Figure 6d). On average the same cCRE overlapped SEACR/Normal peak calls in 3.7 of the 7 tumors (Supplementary Table 1). No SEACR peaks were observed for the kidney sample, as expected given the lack of detectable global or histone locus hypertranscription globally. We conclude that the large majority of strongly RNAPII-hypertranscribed regulatory elements are hypertranscribed in multiple human cancers.

To test whether the variability in hypertranscription between tumors is a consequence of the cell type differences, we obtained 10 μm FFPE tissue sections from a matched liver tumor/normal pair and three additional tumors, all from different patients. RNAPII-Ser5p FFPE-CUTAC revealed that the hypertranscription differences between liver carcinomas from unrelated individuals conspicuously differed. For example, all four cCREs ranked #1 and #2 in either liver tumor showed strong hypertranscription in the first liver tumor but only weak hypertranscription in the second (Figure 6e; Supplementary Figure 5a–c), and similar results were observed for the replication-coupled histone genes (Supplementary Figure 5d). Global hypertranscription for the top-ranked >10,000 cCREs was observed for both liver tumor samples, again much stronger for the first tumor than for the second (Figure 6f). We conclude that the degree of hypertranscription that characterizes a particular human tumor is not cell-type or organ-specific, consistent with our findings of hypertranscription differences between similar mouse brain tumors driven by diverse transgene-encoded factors.

### Probable HER2 amplifications with linkage disequilibrium in human tumors

**Figure 7 |:**
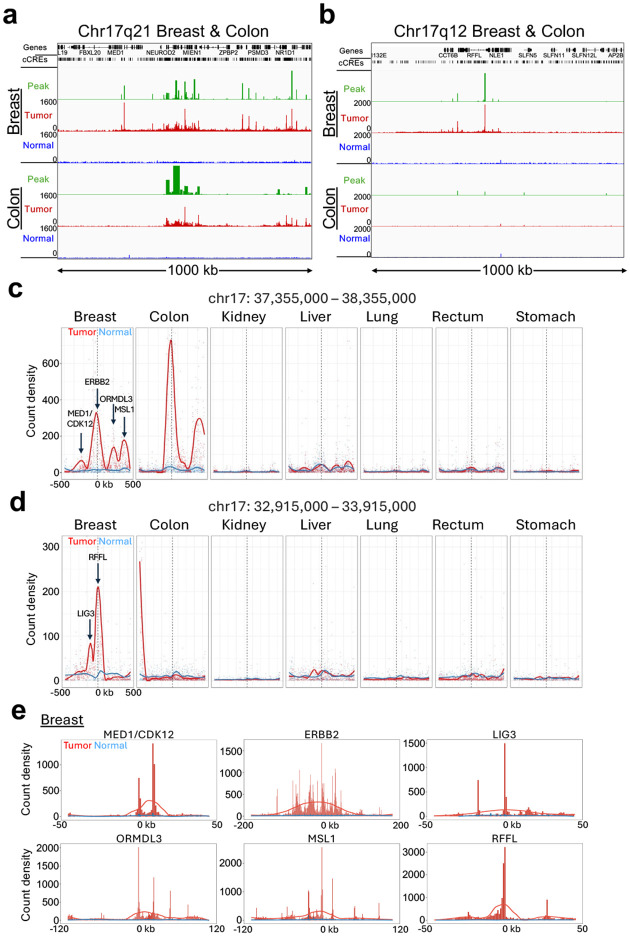
Hypertranscription identifies likely HER2 amplifications and regions of linkage disequilibrium. **a-b** Tracks for the 1-Mb regions on Chromosome 17q21 and 17q12 with the most high-ranking cCREs for both the Breast and Colon samples reveal broad regions of prominent hypertranscription, indicative of likely HER2 amplifications in both tumors. Raw data tracks were group autoscaled together for tumor (red) and normal (blue), where SEACR Tumor peak calls (green) use Normal as the negative control. **c-d**) The two 1-Mb regions displayed in (c-d) were tiled with 1-kb bins and count density curves were fitted for all 7 tumor-normal pairs. Arrows mark the locations of indicated promoter peaks in the breast and colon tumors. **e**) Individual broad summits in (c-d) were zoomed-in and rescaled on *x*-axis centered over the indicated promoter peak and superimposed over raw normalized count tracks scaled to the height of the central peak.

The top-ranked hypertranscribed cCREs overall showed a striking distribution along the genome: Nine of the top 10 and 57 of the top 100 cCREs are located on Chromosome 17 (Supplementary Figure 6a–f; Supplementary Table 1). Most of these cCREs are within either of two contiguous RNAPII-Ser5p-enriched regions of a few hundred kilobases in length in the breast tumor sample not seen in the adjacent normal tissue (Figure 7a–b). These two broad regions of elevated RNAPII correspond to cytological bands Chr17q12 and Chr17q21 and in the case of the colon tumor sample a broad region of RNAPII enrichment is sharply defined in Chr17q21. The breadth of this region sharply localized on Chr17q21 can explain why the colon sample was highly represented on Chromosome 17 based on the ranked cCRE list but showed no hypertranscription at the histone cluster. Major differences between the breast and colon tumors can be seen when sub-regions are group-autoscaled, which identified sharply defined promoter peaks just a few kilobases wide over RFFL, LIG3, ORMDL3 and CDK12 only in the breast tumor and MSL1 and ERBB2 in both the breast and colon tumor (Supplementary Figure 6g–l).

To identify the likely source of regional hypertranscription, we searched PubMed for each of the 100 top-ranked genes with the word “cancer”. This revealed that the most frequently named gene in titles and abstracts by far is ERBB2 in Chr17q21 (35,121 PubMed hits), which accounts for 2/3 of the total, where the next most frequently named gene in the same Chr17q21 region is CDK12 (413 PubMed hits) (Supplementary Table 1). ERBB2 encodes Human Epidermal Growth Factor 2 Receptor (HER2), which is commonly amplified in breast and other tumors and is a target of therapy (24). As our measures of hypertranscription are scaled to the human genome sequence, amplification of a region will appear as a proportional increase in the level of RNAPII over the amplified region, so that we can interpret regional hypertranscription in both our breast and colon tumor samples as revealing independent amplification events.

To identify possible RNAPII hypertranscription features within Chr17q12 and Chr17q21, we tiled 1-kb bins over each 1 Mb region centered on the highest peak in Chr17q21, corresponding to the RFFL promoter in Chr17q12, and the ERBB2 promoter in Chr17q21 and plotted count density within each bin with curve-fitting and smoothing. Remarkably, multiple broad summits appeared in both breast and colon tumor-versus-normal tracks (Figure 7c–d), and the six summits in the breast tumor sample accounted for the six highest ranked Chr17 promoter peaks (Supplementary Figure 6a–f). Of the six summits in the breast sample ERBB2 and MSL1 also appeared in the colon sample, whereas no other tumor samples showed prominent summits above normal in the same region (Figure 7c–d). MSL1 is the human homolog of the Drosophila male-specific-lethal complex, which upregulates male X-chromosome gene expression two-fold; MSL1 encodes a subunit of a histone H4-lysine-16 acetyltransferase complex required for upregulation of the mammalian X chromosome (25). Interestingly, one of the breast summits absent from colon corresponds to the bidirectional promoter of MED1 and CDK12, both of which have been shown to functionally cooperate with co-amplified ERBB2 in aggressive breast cancer (26, 27).

We next superimposed each of the six summits in the Chr17q12–21 region in the breast cancer sample over the raw data tracks on expanded scales for clarity, centered over the highest promoter peak in the region (Figure 7e–f). For ERBB2, the ~100 kb broad summit is almost precisely centered over the ~1 kb wide ERBB2 promoter peak. Although the other summits are less broad, each is similarly centered over a promoter peak, or over the MED1/CDK12 bidirectional promoter peaks. Insofar as there are multiple summits that are one to two orders of magnitude wider than the promoter peaks that they are centered over, our results are inconsistent with independent upregulation of promoters over the HER2 amplified regions. Rather, it would appear that a HER2 amplification event was followed by selection for broad regions around ERBB2 and other loci within each amplicon.

## Discussion

We have shown that hypertranscription at gene regulatory elements can be measured directly using FFPE-CUTAC. Whereas hypertranscription in cancer had been frequently documented in studies based on RNA-seq (2, 3), these indirect methods have limitations owing to variable processing of mRNAs, to the low level of mRNAs encoding critical regulatory proteins and to the need for accurate calibration to genomic DNA abundance. Crucially, none of the methods that have been applied to measure hypertranscription in cancer are suitable for FFPEs, which have long been standard for archival storage of tissue samples (10). Exposure of tissue to ~4% formaldehyde for days badly damages RNA and DNA and causes cross-links to form between tightly bound proteins and nucleic acids. However, this formaldehyde treatment also renders DNA wrapped by lysine-rich histones almost completely refractory, so that open chromatin gaps are the only accessible DNA in the cell. By using antibodies to the phosphorylated RNAPII heptapeptide repeat present in 52 lysine-free tandem copies, FFPE-CUTAC takes advantage of the hyperaccessibility and abundance of the targeted epitope and the intractability of histone cross-linked chromatin to achieve exceptional signal-to-noise. As RNAPII FFPE-CUTAC maps the transcriptional machinery itself directly on the DNA, we obtain ground-truth measurements of transcription, as opposed to inferences based on estimating mRNA abundances. Thus our mapping and quantitation of paused RNAPII, a critical checkpoint between transcriptional initiation and elongation, represents a powerful general approach to characterize hypertranscription at active regulatory elements genome-wide.

To quantify hypertranscription, we used normalized count differences between mouse tumor and normal tissue from the same FFPE section and between matched human tumor and normal tissues (Figure 2a), obviating the need for a spike-in normalization control. First, we mapped Tumor – Normal count differences for ENCODE-annotated cCREs, showing that nearly identical results were obtained regardless of whether the cCRE was a promoter, a proximal or distal enhancer or a CTCF (insulator) site. Next, we confirmed hypertranscription within these samples by examining replication-coupled histone clusters, which serve as proxies for cell proliferation. Finally, we applied a completely unsupervised approach using our SEACR peak-caller to identify hypertranscribed loci throughout the genome. Remarkably, SEACR identified all of the 100 top-ranked of nearly 1 million human cCREs in at least one tumor (Supplementary Table 1), reporting a median of 3.7 overlapping cCREs in six of the seven different human tumors in our study. We also found evidence for reductions in mitochondrial DNA that vary between tumors, suggestive of relaxed selection for oxidative phosphorylation during cancer progression. The rich regulatory information that can be extracted from RNAPII FFPE-CUTAC data using simple analytical tools, despite the use of sparse tissue samples in very poor condition relative to fresh or frozen samples, makes our method especially attractive for application of data mining tools that can be used to infer gene regulatory networks.

Finally, we observed that 55 of the overall top-ranked 100 human cCREs mapped to extensive regions of hypertranscription within Chromosome 17q12–21 in our breast and colon cancer samples. As HER2 amplifications are especially common in breast and colorectal cancer, we infer probable HER2 amplifications, which are known to be subject to clonal selection, resulting in tumor heterogeneity (28). Tumor heterogeneity is consistent with our observation of broad summits centered directly over promoters of candidate cancer driver genes within the amplified regions. Of the six broad summits observed in the breast tumor sample, those centered over ERBB2 and the bidirectional promoters of MED1 and CDK12 were already known to be associated with poor prognosis in HER2-positive breast cancer (26, 27), and MSL1 is part of the complex that upregulates the mammalian X-chromosome (25). Of the other loci identified in this study at peaks in HER2-amplified regions, LIG3 plays a role in DNA repair, so that four of the six loci showing apparent linkage disequilibrium in our breast and colon tumor samples are known or potential cancer biomarkers, consistent with the observation of clonally heterogeneous HER2 amplifications in primary breast tumors by whole-genome sequencing (28). Clonal selection events may be driven by selective sweeps following amplification events that generate extrachromosomal DNA, such as those observed cytologically as double-minute acentric chromosomes that partition unequally during each cell division (29). Our evidence for linkage disequilibrium in the breast and colon samples that we analyzed is consistent with multiple selective sweeps resulting in loss of adjacent but physically unlinked DNA during evolution of these two tumors. Such copy number gains within a tumor can result in tumor heterogeneity and resistance to therapy (30, 31). FFPE-CUTAC thus provides a general diagnostic strategy for detection and analysis of amplifications and clonal selection during cancer progression and therapeutic treatment.

The high signal-to-noise and the abundance of RNAPII and H3K27ac epitopes used in FFPE-CUTAC have made possible detection of global hypertranscription using single 5 μm thick FFPE tissue sections ~1 cm^2^ in area and fewer than 4 million unique fragments. Our identification of probable HER2 amplification and clonal selection events that did not rely on reference to any external data emphasizes the potential power of our approach for understanding basic genetic and epigenetic mechanisms underlying tumor evolution. The simple workflow of FFPE-CUTAC and its potential for scale-up and automation make it an attractive platform for retrospective studies and will require little modification for routine cancer screening and other personalized medicine applications.

## Methods

### Ethical statement

This research was approved by the Fred Hutch Institutional Animal Care and Use Committee (Protocol # 50842) and complies with all required ethical regulations.

### Mice

We used both male and female mice from Jackson lab mouse strain 3529 https://www.jax.org/strain/003529 (FVB/N;C57BL/6;129/Sv). Nestin (N)/tv-a Cdkn2a null pups (P0-P1; male and female) or adults (5–7 week old, male and female) were injected intracranially with either RCAS-PDGFB, RCAS-YAP1-FAM118B, or RCAS-ZFTA-RELA-expressing DF-1 cells and monitored daily for tumor related symptoms for the duration of the experiment. Upon weaning (~P21), mice were housed with same-sex littermates, with no more than 5 per cage and given access to food/water *ad libitum*. All animal experiments were approved by and conducted in accordance with the Institutional Animal Care and Use Committee of Fred Hutchinson Cancer Center (Protocol #50842: Tva-derived transgenic mouse model for studying brain tumors).

### Mouse tumor and normal tissues and FFPEs

Ntva;cdkn2a−/− mice were injected intracranially with DF1 cells infected with and producing RCAS vectors encoding either PDGFB (17), ZFTA-RELA (15), or YAP1-FAM118b (16) as has been described (32). When the mice became lethargic and showed poor grooming, they were euthanized and their brains removed and fixed at least 48 hours in Neutral Buffered Formalin. Tumorous and normal brains were sliced into five pieces and processed overnight in a tissue processor, mounted in a paraffin block and 5- or 10-micron sections were placed on slides. Slides were stored for varying times between 1 month to ~2 years before being deparaffinized and processed for FFPE-CUTAC. Mouse tissue (including normal and tumor bearing brains) were removed, fixed in 10% neutral-buffered formalin for a minimum of 24 hours and embedded into paraffin blocks. 5- or 10-μm serial sections were cut from formalin-fixed paraffin-embedded specimens and mounted on slides.

### Human FFPE slides

The following pairs of human tumor and adjacent normal 5 μm tissue sections from single FFPE blocks were purchased from Biochain, Inc: Breast Normal/Tumor cat. No. T8235086PP/PT; Colon Normal/Tumor cat. No. T8235090PP/PT; Kidney Normal/Tumor cat. No. T8235142PP/PT; Liver Normal/Tumor cat. No. T8235149PP/PT; Lung Normal/Tumor cat. No. T8235152PP/PT; Rectum Normal/Tumor cat. No. T8235206PP/PT; Stomach Normal/Tumor cat. No. T8235248PP/PT. Human primary liver tumor and normal samples were harvested from cases undergoing surgical resection at the University of Washington under the Institutional Review Board approved protocol and then subsequently deidentified.

### Antibodies

Primary antibodies: RNAPII-Ser5p: Cell Signaling Technologies cat. no. 13523, lot 3; RNAPII-Ser2p: Cell Signaling Technologies cat. no. 13499; H3K27ac: Abcam cat. no. ab4729, lot no. 1033973. Secondary antibody: Guinea pig α-rabbit antibody (Antibodies online cat. no. ABIN101961, lot 46671).

### On-slide FFPE-CUTAC

On-slide FFPE-CUTAC was performed as described (11) with modifications. Briefly, FFPE slides were placed in 800 mM Tris-HCl pH8.0 in a slide holder and incubated at 85°C for 1–14 hours, whereupon the paraffin melted and floated off the slide. Slides were cooled to room temperature and transferred to 20mM HEPES pH 7.5,150mM NaCl. Slides were drained and excess liquid wicked off using a Kimwipe tissue. The sections were immediately covered with 20–60 μL primary antibody in Triton-Wash buffer (20mM HEPES pH 7.5,150mM NaCl, 2mM spermidine and Roche complete EDTA-free protease inhibitor) added dropwise. Plastic film was laid on top to cover and slides were incubated ≥2 hr incubation at room temperature (or overnight at ~8°C) in a moist chamber. The plastic film was peeled back, and the slide was rinsed once or twice by pipetting 1 mL Triton-Wash buffer on the surface, draining at an angle. This incubation/wash cycle was repeated for the guinea pig anti-rabbit secondary antibody (Antibodies Online cat. no. ABIN101961) and for pAG-Tn5 preloaded with mosaic end adapters (Epicypher cat. no. 15–1117 1:20), followed by a Triton-Wash rinse and transfer of the slide to 10 mM TAPS pH 8.5. Tagmentation was performed in 5mM MgCl_2_, 10mM TAPS pH 8.5, 20% (v/v) N,N-dimethylformamide in a moist chamber and incubated at 55°C for 1 hr. Following tagmentation, slides were dipped in 10 mM TAPS pH 8.5, drained and excess liquid wicked off. Individual sections were covered with 2 μL 10% Thermolabile Proteinase K (TL ProtK) in 1% SDS using a pipette tip to loosen the tissue. Tissue was transferred to a thin-wall PCR tube containing 2 μL TL ProK using a watchmaker’s forceps, followed by 1 μL TL ProtK and transfer to the PCR tube. Tubes were incubated at 37°C for 30 min and 58°C for 30 min before PCR as described above.

### FFPE-CUTAC for curls

Curls were transferred to a 1.7 mL low-bind tube (Axygen cat. no. MCT-175-C), which tightly fits a blue pestle (Fisher cat. on. 12–141-364). Mineral oil (200 μl) was added and the tube was placed in a 85–90°C water bath for up to 5 min to melt the paraffin. The suspension was then homogenized ~10–20 sec with a pestle attached to a pestle motor (DWK Life Sciences cat no. 749540–0000). Warm cross-link reversal buffer (200 μl 800 mM Tris-HCl pH8.0) was added followed by addition of 6 μl of 1:10 Biomag amine paramagnetic beads (48 mg/ml, Polysciences cat. no. 86001–10). Homogenization was repeated, and 800 μl warm cross-link reversal buffer was added. Tubes were incubated at 85–90°C for 1–14 hours, vortexed, centrifuged briefly and the mineral oil was removed from the top without disturbing the surface. A 500 μl volume of mineral oil was added, mixed by inversion, centrifuged and the mineral oil removed leaving a thin oil layer. A 2.4 μl volume of agarose glutathione paramagnetic beads (Fisher cat. no. 88822) was added below the surface and mixed by inversion on a Rotator. Tubes were centrifuged briefly, placed on a strong magnet (Miltenyi Macsimag separator, cat. no. 130–092-168), and the supernatant removed and discarded, and the bead-bound homogenate was resuspended in up to 1 mL Triton-wash buffer (20 mM HEPES pH 7.5, 150 mM NaCl, 0.5 mM spermidine, 0.2mM EDTA, 0.05% Triton-X100 and Roche EDTA-free protease inhibitor) and divided into PCR tubes for antibody addition. Other steps through to library preparation and purification followed the standard FFPE-CUTAC protocol (11). Detailed step-by-step protocols for slides and curls is available on Protocols.io: https://www.protocols.io/edit/cutac-for-ffpes-c5huy36w.

### DNA sequencing and data processing

The size distributions and molar concentration of libraries were determined using an Agilent 4200 TapeStation. Barcoded CUT&Tag libraries were pooled at equal volumes within groups or at approximately equimolar concentration for sequencing. Paired-end 50×50 bp sequencing on the Illumina NextSeq 2000 platform was performed by the Fred Hutchinson Cancer Research Center Genomics Shared Resources.

### Data analysis

#### Preparation of the CCREs

We obtained the mm10 and hg38 versions of the Candidate cis-Regulatory Elements by ENCODE (https://screen.encodeproject.org/) from UCSC (33). For mouse mm10 we used all 343,731 entries. Because our sequencing data was aligned to hg19, we used UCSC’s liftOver tool to reposition the hg38 CCREs resulting in 924,834 entries. We noticed that many human CCREs were in repeated regions of the genome so we intersected the hg19 CCRE file with UCSC’s RepeatMasked regions using bedtools 2.30.0 (34) “intersect -v” command to make a file of 464,749 CCREs not in repeated regions.

#### Preparation of histone regions

For mm10 we used these regions:

chr13 21715711 21837530 H2bc13-H4bc2

chr13 22035122 22043658 H2ac12-H2bc11

chr13 23531044 23622558 H4c8-H1f4

chr13 23683473 23764412 H2ac6-H1f1

For hg19 we used these regions:

chr1 149783434 149859466 Minor

chr6 26017260 26285727 Major

#### Alignment of PE50 Illumina sequencing

1. We used cutadapt 2.9 (35) with parameters “-j 8 --nextseq-trim 20 -m 20 -a AGATCGGAAGAGCACACGTCTGAACTCCAGTCA -A AGATCGGAAGAGCGTCGTGTAGGGAAAGAGTGT -Z” to trim adapters from 50bp paired-end reads fastq files.

2. We used Bowtie2 2.4.4 (36) with options “--very-sensitive-local --soft-clipped-unmapped-tlen --dovetail --no-mixed --no-discordant -q --phred33 -I 10 -X 1000” to map the paired-end 50bp reads to the mm10 Mus musculus or hg19 Homo sapiens reference sequences obtained from UCSC.

3. We used samtools 1.14 (37) “view” to extract properly paired reads from the mm10 alignments into bed files of mapped fragments.

4. We computed the fraction of fragments mapped to chrM.

5. We used bedtools 2.30.0 “genomecov” to make a normalized count track which is the fraction of counts at each base pair scaled by the size of the reference sequence so that if the counts were uniformly distributed across the reference sequence there would be one at each position.

6. We ran Picard 2.18.29 (38) MarkDuplicates program on the sam output from bowtie2.

#### Preparation of aligned samples

1. For mouse, we used all mapped fragments. For human, we removed duplicates as marked by Picard from the sam files before making normalized count tracks.

2. For mouse on-slide experiments, we merged tumor replicates within the experiment. For human, we merged mapped fragments from 5 different experiments for each tumor and then equalized the numbers of fragments for tumor and normal pairs by downsampling the larger of the two using the UNIX shuf command.

#### Peak-finding

We ran SEACR 1.3 (18) with parameters “norm relaxed” on tumor samples with the normal sample from each tumor and normal pair as the control. For comparison, we also called peaks after reversing the roles of tumor and normal.

#### Preparation of the per-CCRE and per-Histone region files

We used the bedtools intersect and groupby commands to sum the number of normalized counts from the tracks within the cCRE and histone region boundaries. Because the cCREs and histone regions vary in size, we then averaged the number of normalized counts within each to make them more comparable. The resulting files have one row per cCRE or histone region and one column per sample and are suitable for submission to the Degust server (https://degust.erc.monash.edu/) using the Voom/Limma option (-log_10_FDR versus log_2_FoldChange).

#### Preparation of Tumor-Normal files

We computed Tumor-Normal pairs from the CCRE region files and sorted them by largest differences in absolute value (Supplementary Table 1).

#### Curve-fitting

We partitioned the genome into 1 kb tiles and merged replicates, then downsampled to equalize library sizes between tumor and normal samples from each patient and added up normalized counts within each tile. For each tumor and normal patient sample, we fit t he n ormalized counts across tiles using a Local Polynomial Regression (LOESS) model as implemented in the `stats` package of the R programming language, setting the degree of smoothing to span = 0.2 (Figure 7c–d) or 0.5 (Figure 7e).

### Statistics and Reproducibility

No statistical method was used to predetermine sample size nor were data were excluded from the analyses. The experiments were not randomized and Investigators were not blinded to allocation during experiments and outcome assessment.

## Supplementary Material

Supplement 1

1

## Data Availability

The sequencing data generated in this study have been deposited in the NCBI GEO database under accession code GSE261351.
