# RNA Polymerase II hypertranscription in cancer FFPE samples

**DOI:** 10.1101/2024.02.28.582647

**Published:** 2024-05-21

**Authors:** Steven Henikoff, Jorja G. Henikoff, Ronald M. Paranal, Jacob E. Greene, Ye Zheng, Zachary R. Russell, Frank Szulzewsky, Sita Kugel, Eric C. Holland, Kami Ahmad

**Affiliations:** 1 Basic Science Division, Fred Hutchinson Cancer Center, Seattle, WA, USA; 2 Howard Hughes Medical Institute, Chevy Chase, MD, USA; 3 Human Biology Division, Fred Hutchinson Cancer Center, Seattle, WA, USA; 4 Molecular Medicine and Mechanisms of Disease (M3D) PhD Program, University of Washington, Seattle, WA, USA; 5 Vaccine and Infectious Disease Division, Fred Hutchinson Cancer Center, Seattle, WA, USA

**Keywords:** Gene Regulation, Epigenomics, CUT&Tag, Mitochondrial DNA, Histone genes, HER2 amplification, CDK12 cyclin-dependent kinase

## Abstract

Hypertranscription is common in human cancers and predicts poor prognosis. However detection of hypertranscription is indirect, relying on accurately quantifying mRNA levels and estimating cell numbers. Previously, we introduced FFPE-CUTAC, a genome-wide method for mapping RNA Polymerase II (RNAPII) in formalin-fixed paraffin-embedded (FFPE) sections. Here we use FFPE-CUTAC to demonstrate genome-wide hypertranscription both in transgene-driven mouse gliomas and in assorted human tumors at active regulatory elements and replication-coupled histone genes with reduced mitochondrial DNA abundance. FFPE-CUTAC identified RNAPII-bound regulatory elements shared among diverse cancers and readily categorized human tumors despite using very small samples and low sequencing depths. Remarkably, RNAPII FFPE-CUTAC identified *de novo* and precisely mapped HER2 amplifications punctuated by likely selective sweeps including genes encoding direct positive regulators of RNAPII itself. Our results demonstrate that FFPE-CUTAC measurements of hypertranscription and classifications of tumors using small sections provides an affordable and sensitive genome-wide strategy for personalized medicine.

## Introduction

Global increase in nascent transcription, referred to as hypertranscription, is a general feature of cells undergoing proliferation during development (1) and has been extensively documented in cancer (2, 3). For example, high-level expression of the *cMyc* oncogene has been hypothesized to act as a transcriptional amplifier (4-6), globally increasing the frequency of transcriptional bursting at promoters of active genes within mammalian genomes (7). Genome-wide hypertranscription has also been observed more generally in aggressive human cancers under control of a wide variety of transcription factors (TFs) in addition to *cMyc* (2). Hypertranscription in cancer has been attributed to widespread loss of transcriptional repression (2), perhaps due to an inability of topoisomerase I to prevent transcription overdrive (9).

Studies of genome-wide hypertranscription in cancer have relied on measurements of abundant stable mRNAs, but scaling the level of any particular mRNA to the level of its template DNA is challenging. Most hypertranscription estimates have been based on calibrating RNA-seq data using spike-ins (6), although estimates have also been based on polymorphisms in regions of cancer-specific loss of heterozygosity (2), on estimates of DNA abundance and ploidy in matched RNA-seq and DNA-seq samples (3) or on single-cell mRNA abundances (10). However, these estimates based on RNA-seq measurements are complicated by variability in mRNA lifetimes and are relatively insensitive to low-level transcripts such as those for transcription factors that drive development and are misregulated in cancer. Thus far, detection of hypertranscription has relied on high-quality RNA-seq data, for example from freshly cryopreserved patient samples (11). However, the vast majority of patient samples are fixed in formalin and embedded in paraffin, a procedure that has been the standard for well over a century (12). The days-long fixation in formalin (~4% formaldehyde) results in cross-linking and adduct formation on both RNA and DNA, and although commercial FFPE extraction and sequencing kits have been developed, they have not been used to detect hypertranscription.

Previously, we showed that formalin treatment could be used to our advantage by profiling the RNA Polymerase II (RNAPII) transcriptional machinery, providing a direct genome-wide map of transcription on the DNA itself (13). Specifically, we had modified our antibody-directed Cleavage Under Targets and Tagmentation (CUT&Tag) *in situ* chromatin profiling method such that for promoter and enhancer epitopes, tethered Tn5 transposase would integrate DNA sequencing adapters into nearby open chromatin regions under low-salt conditions (14). Our CUTAC (Cleavage Under Targeted Accessible Chromatin) method mapped open chromatin with especially high resolution and signal-to-noise, with best results obtained using antibodies to RNA Polymerase II (RNAPII) serine phosphate epitopes (15, 16). Our modification of CUTAC for FFPEs (FFPE-CUTAC) allowed for high-confidence identification of known biomarkers in mouse brain cancers, including microRNA tumor suppressor loci invisible to standard RNA-seq. FFPE-CUTAC using both RNAPII-Ser5p and histone H3K27ac antibodies also provided much better biomarker discrimination than RNA-seq on fresh mouse brain tumor samples.

Here we show that FFPE-CUTAC can be used to directly map hypertranscription at regulatory elements throughout the mouse genome, revealing that the degree of hypertranscription varies between genetically identical tumors and for some is not observed at all. When applied to tumor and adjacent normal 5 micron ~1 cm^2^ human FFPE sections from seven anonymous individual human tumors, FFPE-CUTAC analyzed for hypertranscription identified dozens of strongly hypertranscribed loci in common among the tumors. Strikingly, in two of the seven individual tumors we observed broad increases of RNAPII within Chromosome 17q12-21, which includes the *ERBB2* (*HER2*) locus. These evident *HER2* amplifications were punctuated with broad hypertranscribed regions, suggestive of linkage disequilibrium during tumor evolution (17, 18). Our data suggest that selective sweeps of direct regulators of RNAPII, including the CDK12 kinase, contribute to the poor prognosis associated with hypertranscription. The ability of FFPE-CUTAC to categorize tumors with sparse material, precisely localize patterns of regulatory element hypertranscription, and map megabase-sized regions of amplification interspersed with smaller regions of likely clonal selection, makes it an attractive platform for general personalized medicine applications.

## Results

### Hypertranscription in mouse brain tumors

In our earlier FFPE-CUTAC study (13), we observed that significantly upregulated cCREs were more frequent than downregulated cCREs in mouse brain tumors with different transgene drivers: a ZFTA-RELA (RELA) transcription factor gene fusion driving an ependymoma (19), a YAP1-FAM118b (YAP1) transcriptional co-activator gene fusion driving an ependymoma (20), and overexpression of the tyrosine-kinase active PDGFB ligand driving a glioma (21). Upregulation bias based on RNAPII log_2_(fold-change) plotted on the *y*-axis as a function of log_10_(average signal) on the *x*-axis (MA plot) is observed in pooled data from several experiments in which tumor-rich sections were separated from normal sections (Figure S1a-e). The upregulation bias is much greater for RELA than for PDGFB, and to further understand these differences and to eliminate sample-to-sample variability, we examined on-slide dissection data from single FFPE slides representing normal mouse brain, RELA and YAP1 tumors and PDGFB tumors from two genetically identical mice. Unexpectedly, upregulation based on fold-change showed little relationship to the percentage of tumor in the sample as determined by counting cells stained for tumor transgene expression. For example, YAP1 tumor sections averaged 16% tumor cells and showed similar upregulation bias to the PDGFB-1 sections with 80% tumor cells and stronger upregulation bias than the PDGFB-2 sections with 64% tumor cells (Figure S1f-h, right panels) and all three showed weaker upregulation versus the RELA tumor sections with 40% tumor cells (Figure S1f-h, left panels). The fold-change ratio of tumor:normal does not distinguish between a weak signal increasing to moderate strength and a moderate signal increasing to high strength. However, we noted that significant upregulation differences increased in log_10_(RNAPII) signal with reduced fold-change (red dots approaching the *x*-axis left-to-right), which suggests that the major differences in RNAPII upregulation in tumors result from significant increases in already high transcription levels, *i.e.* hypertranscription.

**Figure 1 ∣:**
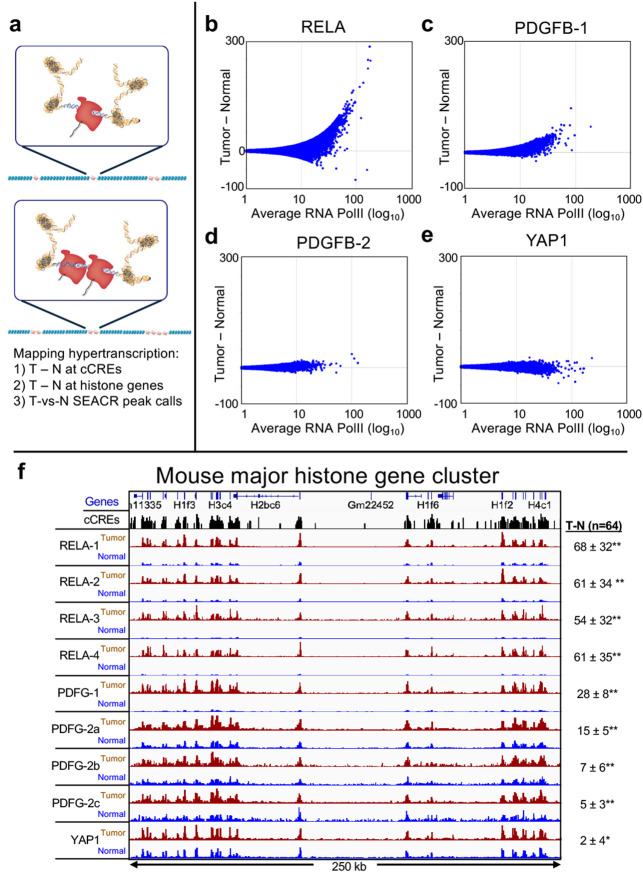
RNAPII-Ser5p FFPE-CUTAC directly maps hypertranscription. **a)** Model for hypertranscription in cancer: Paused RNA Polymerase II (RNAPII) at active gene regulatory elements such as promoters and enhancers increases on average over the cell cycle resulting in a net proportional gain in RNAPII occupancy across the genome. Using RNAPII FFPE-CUTAC we can map hypertranscription genome-wide using three complementary approaches: 1) Genome-scaled Tumor (T) minus Normal (N) counts at cCREs, 2) T – N at replication-coupled histone genes and 3) SEACR Tumor peak calls using Normal as the background control. **b-e**) Bland-Altman plots showing hypertranscription mapped over the 343,731 annotated mouse cCREs for tumor and normal sections dissected post-tagmentation from a 10 micron FFPE slice from each of four different paraffin blocks. Hypertranscription of a cCRE is defined as the excess of RNAPII-Ser5p in the indicated tumor over normal (Tumor minus Normal in normalized count units for Mm10-mapped fragments pooled from the same slide). **f**) Hypertranscription at replication-coupled histone genes. Slides used for PDGFB-2a-c were from the same paraffin block but used in different experiments, and all others were from different paraffin blocks. Numbers at right were obtained by subtracting the sum of normalized counts in the normal sections from that in the tumor sections over all 64 annotated single-exon replication-coupled histone genes, where the Standard Deviation is shown. Paired *t*-test: * *p* < 0.001; ** *p* < 0.00001.

Our RNAPII FFPE-CUTAC assay is well-suited to detect minor absolute differences in regulatory element RNAPII occupancy (Figure 1a), unlike RNA readouts that require calibration to the DNA template. For each tumor and normal sample, we counted the number of mapped fragments spanning each base-pair in a cCRE scaled to the mouse genome and averaged the number of counts over that cCRE. To sensitively detect hypertranscription, we plotted Tumor minus Normal counts on the *y*-axis versus average RNAPII signal (Bland-Altman plot (22)) plotted on a log_10_ scale on the *x*-axis for clarity. This revealed clear hypertranscription for RELA (Figure 1b). Interestingly, PDGFB tumors differed in hypertranscription, strongly in PDGFB-1 (Figure 1c) and very weakly in PDGFB-2 (Figure 1d), whereas YAP1 showed weak hypotranscription. To determine whether hypertranscription assayed by RNAPII abundance over cCREs is specific to any particular class of regulatory element(s), we divided up the data presented in Figure 1 into the five ENCODE-annotated categories: Promoters (24,114), H3K4me3-marked cCREs (10,538), Proximal Enhancers (108,474), Distal Enhancers (211,185) and CTCF cCREs (24,072). We observed that the five RNAPII hypertranscription profiles are highly consistent with one another (Figure S2), which suggests that RNAPII abundance differences between tumors and normal brain affect all regulatory element classes.

To verify that these differences in global upregulation of cCREs are related to tumor growth, we examined the profiles of the replication-coupled histone genes, which provides an independent measure of cell proliferation. In total, these small single-exon genes produce RNAPII-dependent U7-processed single-exon mRNAs during S-phase to encode for the histones that package the entire genome in nucleosomes, and so the abundance of RNAPII at these histone gene loci provides a proxy for steady-state DNA synthesis genome-wide. Of the 64 mouse replication-coupled genes, 54 are within the major histone gene cluster on Chromosome 13, and when Tumor and Normal dissection data from multiple experiments are displayed, we see differences between tumor samples consistent with our observation of RNAPII hypertranscription differing between samples (Figure 1f). For quantitative validation, we calculated the excess of normalized counts for each experiment, with strongly significant increases for the four RELA and three PDGFB-2 biological replicates and for PDGFB-1, with a small weakly significant increase for YAP1. The consistency between our measurements of hypertranscription over gene regulatory elements and S-phase-dependent hypertranscription over histone loci confirm that hypertranscription is a real, but variable tumor-specific property of transgene-driven mouse tumors. As these exceptionally S-phase-dependent histone loci are expressed in proportion to the amount of replicated DNA (23), we suggest that cancer cells increase engaged RNAPII at these loci to load up on histones at S-phase for increased cell proliferation.

### Hypertranscription varies between human tumors

**Figure 2 ∣:**
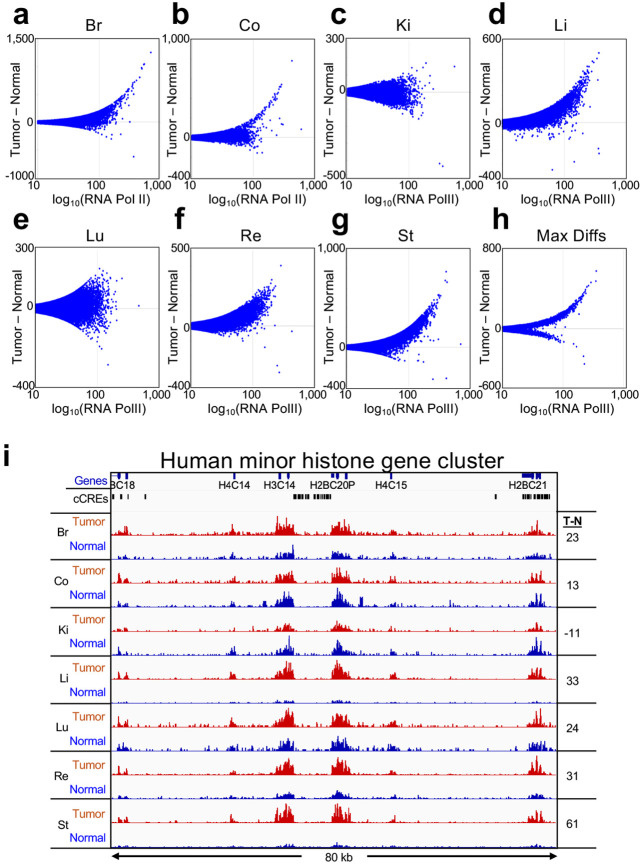
Hypertranscription in human Tumor-vs-Normal tissues: **a-h**) All fragments were pooled from four slides from the same paraffin block and the number of fragments equalized between tumor and normal for each of the seven cancers. Bland-Altman plots showing hypertranscription mapped over the 984,834 annotated mouse cCREs for tumor and matched normal sections from 5 micron FFPE slices. Max Diffs displays the Tumor minus Normal maximum of the seven samples for each cCRE. **i**) The minor human histone gene cluster on Chr 1 is shown, where tracks are autoscaled for each Tumor (red) and Normal (blue) pair. As individual samples are not intended to represent tumor types, sample names are abbreviations (Figure S3).

To expand on our findings of hypertranscription based on transgene-driven mouse brain tumors to a diverse sample of naturally occurring cancers, we obtained 5 μm FFPE sections on slides prepared from paraffin blocks of anonymous human tumor and adjacent normal pairs (Figure S3). We performed RNAPII-Ser5p FFPE-CUTAC and rank-ordered each pair by Tumor minus Normal differences to test for RNAPII hypertranscription based on the 984,834 ENCODE-annotated human cCREs. To avoid possible imbalances in the comparisons between tumor and normal pairs, we removed cCREs in repeat-masked regions of the hg19 build, pooled the data from all four independent experiments and equalized the number of fragments between tumor and normal samples. We observed clear hypertranscription in five of the seven samples (Br, Co, Li, Re and St) and for the composite of all samples (Figure 2a-h). In contrast, the Ki and Lu tumors showed essentially no hypertranscription. To evaluate the robustness of these hypertranscription results, we plotted hypertranscription for the data from a single slide for each specimen, and despite sparse data owing to the ~1 cm^2^ size of the 5 μm sections we observed very similar results (Figure S4a-p). We also obtained very similar results when we removed duplicates and equalized the number of fragments for each tumor-normal pair (Figure S4q-x).

As was the case for the mouse tumors, hypertranscription could be observed by examining the human replication-coupled histone loci. Although the data were relatively sparse, our Br, Li, Re and St cancer samples showed prominent hypertranscription over the ~80-kb region spanning the human minor histone cluster whereas the Ki sample showed hypotranscription and the Lu sample showed little if any difference in RNAPII abundance (Figure 2i). Unexpectedly, our Co cancer sample showed no detectable difference between tumor and normal at the histone loci, despite the strong hypertranscription over cCREs. Together, our results suggest that FFPE-CUTAC can sensitively measure hypertranscription in small sections of the type that are routinely used by pathologists for cytological staining and analysis.

**Figure 3 ∣:**
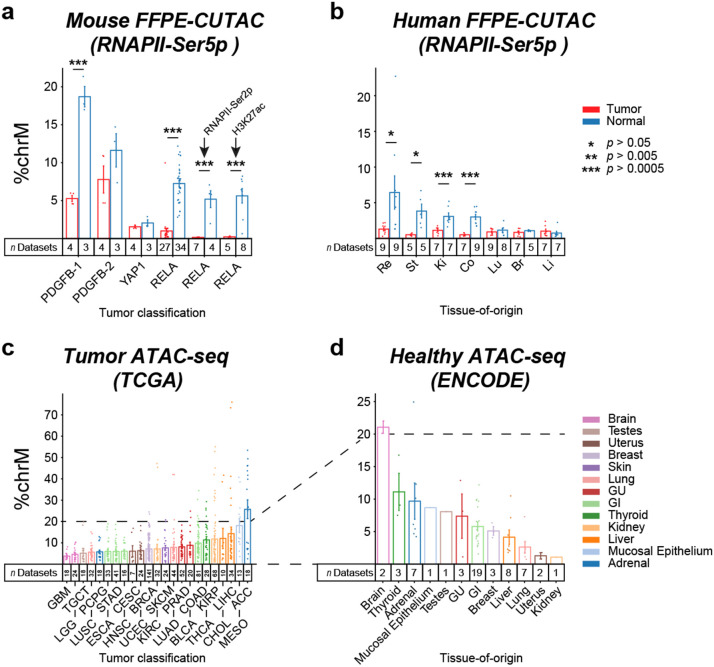
FFPE-CUTAC mitochondrial DNA signal is reduced in tumors. **a**) The percentage of normalized counts mapping to Chromosome M (ChrM = mitochondrial DNA) was calculated for FFPE-CUTAC data from four mouse brain tumor paraffin blocks driven by PDGFB, YAP1 and RELA transgenes. An RNAPII-Ser5p antibody was used for the first four comparisons, and an RNAPII-Ser2p and histone H3K27ac antibodies were used respectively for the fifth and sixth comparisons. **b**) Same as (a) for RNAPII-Ser5p FFPE-CUTAC data for the seven human Tumor/Normal pairs used in this study. **c-d**) ATAC-seq count data from TCGA (tumor) and ENCODE (normal) shows variability in ChrM percentages between tumors, consistent with our finding based on FFPE-CUTAC.

FFPE-CUTAC and other tagmentation methods non-specifically recover a small fraction of mitochondrial DNA (mtDNA, Chromosome M) due to the enhanced accessibility of nucleosome-free mtDNA. However, RNAPII-Ser5p FFPE-CUTAC detected a much lower level of mtDNA in most tumor samples than in their matched normal samples for both mouse and human (Figure 3a-b), suggesting that these tumors contain fewer mitochondrial genomes. To test this interpretation, we mined publicly available ATAC-seq data from both the TCGA and ENCODE projects. In the case of TCGA tumor data, the percentage of mtDNA ranges from ~4% for glioblastoma, a brain cancer, to ~25% for adrenal carcinoma, whereas for ENCODE data, which are from healthy individuals, percentages range from ~1% for kidney to ~21% for brain (Figure 3c-d). This 6-fold higher level of mitochondrial ATAC-seq signal in normal brain in the ENCODE data over that of glioblastoma in the TCGA data is consistent with decreased mitochondrial DNA abundance in most human and mouse tumors in our FFPE-CUTAC data. These reductions in mtDNA by both CUTAC and ATAC-seq are consistent with reductions in mtDNA reported based on whole-genome sequencing (24), suggestive of relaxed selection for maintenance of mtDNA in cancer.

### Most hypertranscribed regulatory elements are shared between diverse human cancers

We wondered whether our observations of hypertranscription in cancer based on annotated cCREs and histone genes could be generalized using an approach that does not depend on annotations of any kind. Previously, our lab introduced SEACR (Sparse Enrichment Analysis for CUT&RUN), which was designed for application to low read-count data (25). SEACR optionally uses a background control dataset, typically for a non-specific IgG antibody. To customize SEACR for hypertranscription in cancer, we replaced the background control with the normal sample in each pair, merged fragment data, removed duplicates and equalized read numbers for our seven human Tumor/Normal pairs. SEACR reported a median of 4483 peaks, and when Tumor and Normal were exchanged, a median of only 15 peaks was reported, which suggests that hypertranscription is more common than hypotranscription. Therefore, we can use SEACR Tumor/Normal peaks as an unbiased method for discovering the most hypertranscribed loci in our human cancer samples.

**Figure 4 ∣:**
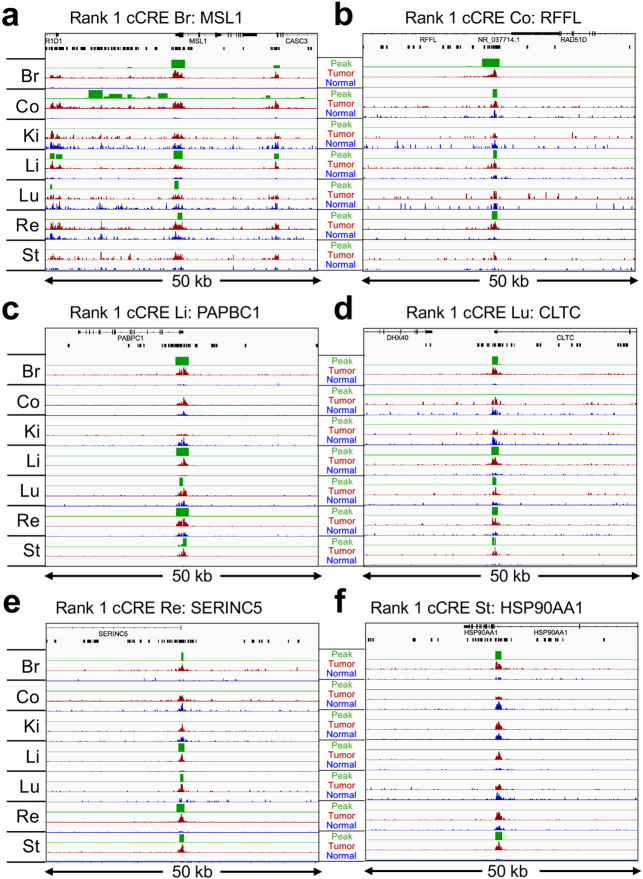
Top-ranked human cCREs based on hypertranscription correspond to SEACR Tumor-vs-Normal RNAPII-Ser5p peaks. For each of the indicated tumors, tracks are shown for 50-kb regions around the #1-ranked cCRE based on Tumor (dark red) and Normal (blue) counts. Raw data tracks were group-autoscaled together for tumor (red) and normal (blue), where SEACR Tumor peak calls (green) use Normal as the negative control. Gene annotations and cCREs (black rectangles) are shown at top.

We first asked whether SEACR Tumor/Normal peak calls corresponded to the 100 top-ranked cCREs in the overall list representing all seven tumors. Remarkably, all 100 cCREs at least partially overlapped one or more SEACR Tumor/Normal peak call, and in addition, the large majority of the 100 top-ranked cCREs intersected with overlapping SEACR peak calls from multiple Tumor/Normal pairs (Supplementary Table 1). Each of the #1-ranked cCREs in the Br, Co, Li, Lu and Re tumor samples respectively intersected MSL1, RFFL, PABPC1, CLTC and SERINC5 genes and also overlapped SEACR peak calls in 4-5 of the 7 tumors (Figure 4a-e). Additionally, the #1-ranked cCRE in the St sample intersected an intergenic enhancer in the HSP90AA1 gene and overlapped SEACR peak calls in both Br and St (Figure 4f). On average the same cCRE overlapped SEACR/Normal peak calls in 3.7 of the 7 tumors (Supplementary Table 1). No SEACR peaks were observed for the kidney sample, as expected given the lack of detectable cCRE or histone locus hypertranscription. We conclude that the large majority of strongly RNAPII-hypertranscribed regulatory elements are hypertranscribed in multiple human cancers.

**Figure 5 ∣:**
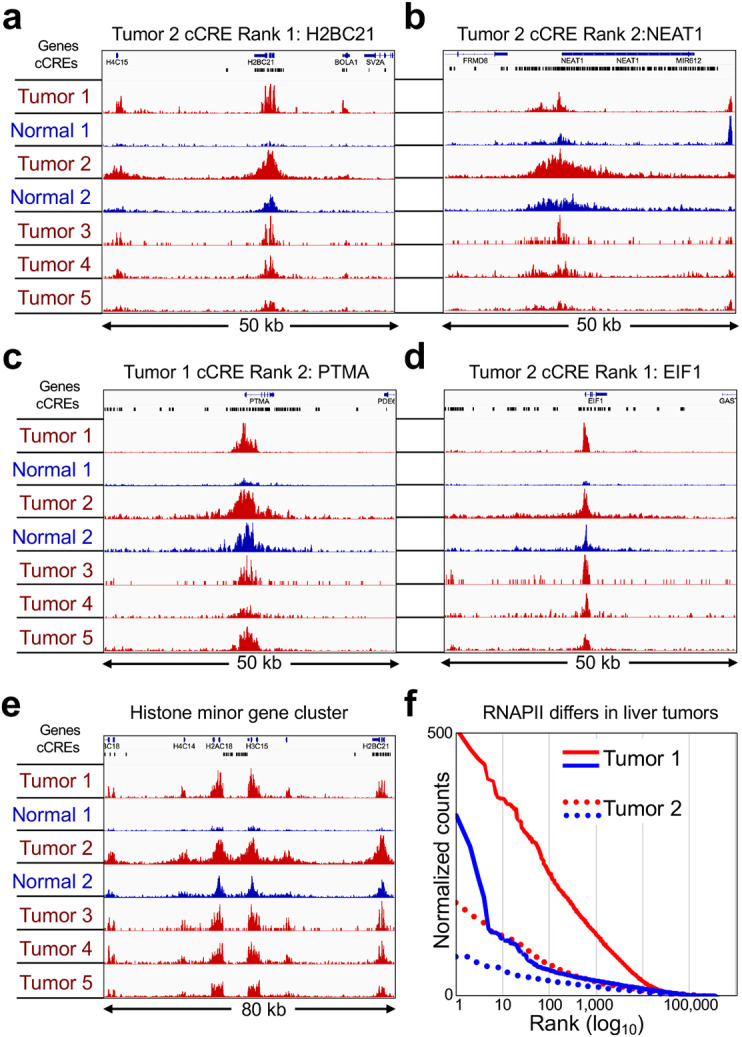
Hypertranscription differs between human liver tumors. **a-d**) Top-ranked cCREs based on liver tumors 1 and 2 (red) and matched normal (blue) counts. Tumor/Normal tracks and Tumors 3-5 are group-autoscaled. **e**) Same as (a), except for the minor histone gene cluster on Chromosome 1. **f)** Levels of hypertranscription differ between different hepatocarcinomas (Tumor 1: solid lines, Tumor 2 dotted lines, where tumor is red and normal is blue). For each tumor and normal sample, we counted the number of mapped fragments spanning each base-pair in a cCRE scaled to the human genome and averaged the number of counts over that cCRE. We rank-ordered based on tumor minus normal representing global upregulation, and conversely rank-ordered cCREs based on normal minus tumor representing global downregulation. With such a large collection of loci, our a priori expectation is that the rank-ordered distribution of differences between tumor and normal will be approximately the same regardless of whether the differences are based on tumor minus normal or normal minus tumor. For clarity, we plotted rank-ordered differences on a log_10_ scale.

To test whether cell-type differences in hypertranscription can account for variability between human tumors in our sample, we obtained 10 μm FFPE tissue sections from a matched liver tumor/normal pair and three additional liver tumors, all from different patients. RNAPII-Ser5p FFPE-CUTAC revealed that the hypertranscription differences between liver tumors from unrelated individuals conspicuously differed. For example, all four cCREs that ranked #1 and #2 in either liver tumor showed strong hypertranscription in the first liver tumor but only weak hypertranscription in the second (Figure 5a-d), and similar results were observed for the replication-coupled histone genes (Figure 5e). Hypertranscription for the top-ranked >10,000 cCREs was observed for both liver tumor samples, again much stronger for the first tumor than for the second (Figure 5f).

### Sparse FFPE-CUTAC data resolves tumor diversity

**Figure 6 ∣:**
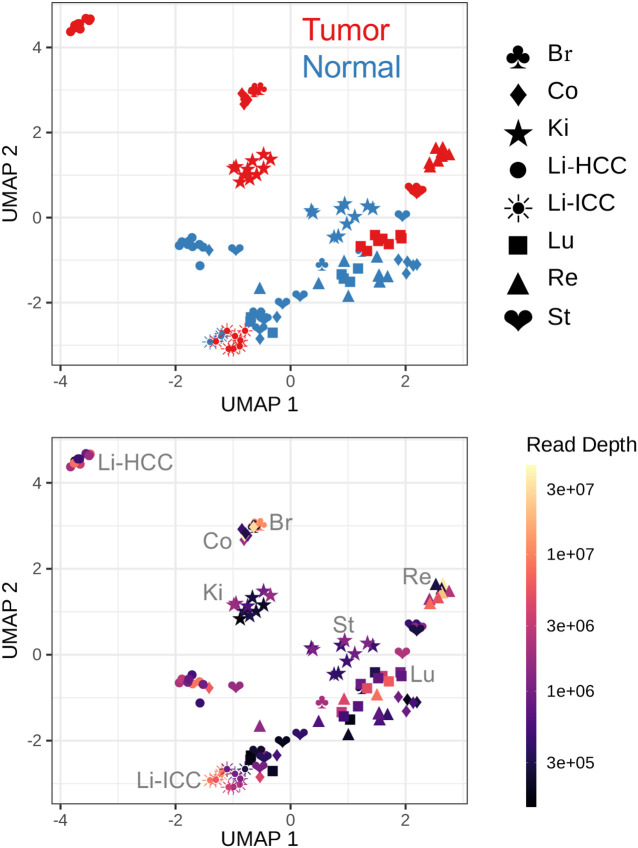
Tight clustering of tumor samples. **a**) UMAP of 114 human tumor samples. **b**) Same as (a) except colored for sequencing depth and indicating homogeneous tumor clusters.

The identification of so many of the top hypertranscribed loci shared by multiple individual tumors raised the question of how effectively FFPE-CUTAC can resolve differences between cancers. Accordingly, we constructed a cCRE-based UMAP including all 114 individual human samples with >100,000 mapped fragments (median 925,820). Whereas normal samples produced loose clusters with some mixing, tumor samples formed tight homogeneous clusters separated by cell type (Figure 6a). This implies that paused RNAPII at regulatory elements is more discriminating between tumors than between the tissues that the tumors emerge from. Relatively few samples and shallow sequencing depths were needed for tight clustering; for example, the stomach tumor cluster comprised four samples from four different experiments with a median of ~470,000 mapped fragments (Figure 6b and Supplementary Information). Interestingly, the Co and Br tumor samples clustered very close to one another, suggesting that this pair of individual tumors share oncogenic loci to a much greater extent than would be expected for such different tissue types.

### HER2 amplifications with linkage disequilibrium in human tumors

**Figure 7 ∣:**
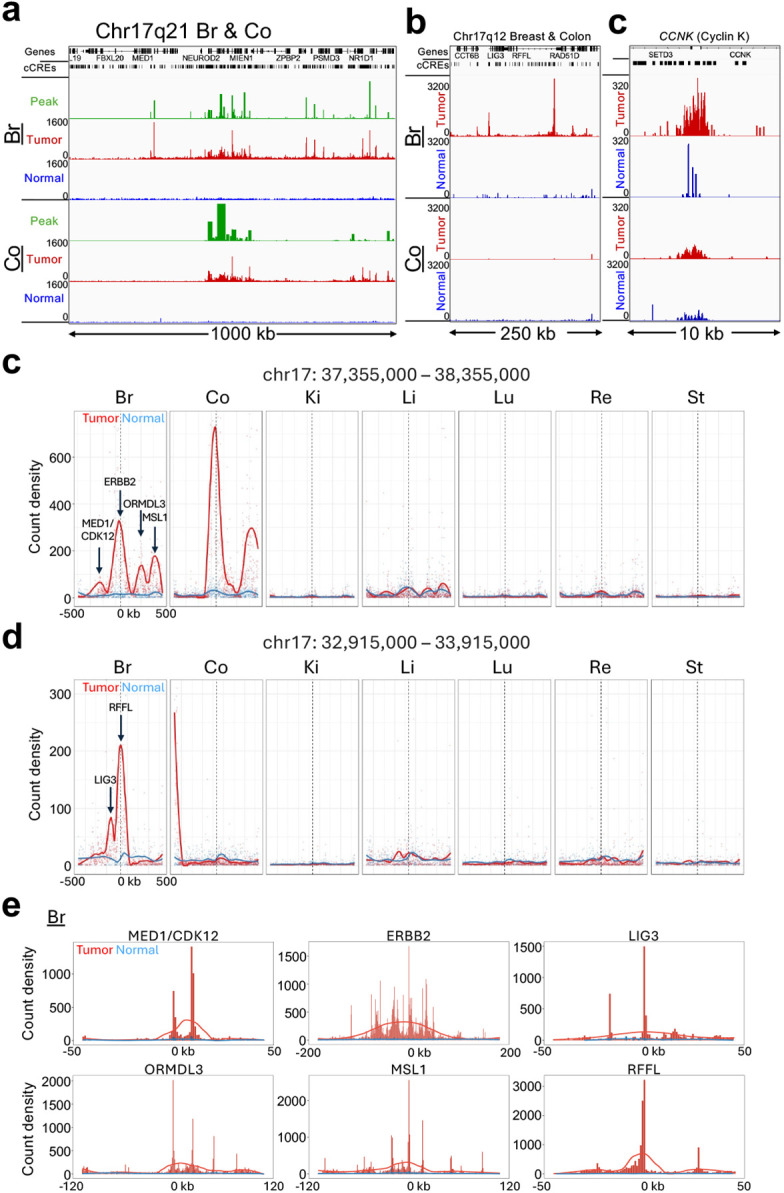
Hypertranscription identifies likely HER2 amplifications and regions of linkage disequilibrium. **a**) Raw data tracks for the 1-Mb region on Chromosome 17q21 were group-autoscaled together for tumor (red) and normal (blue), where SEACR Tumor peak calls (green) use Normal as the negative control. Broad regions of prominent hypertranscription, indicate likely HER2 amplifications in both tumors. **b**) Raw data tracks for the 250-kb 17q12 region amplified in Br but evidently not in Co. **c**) Raw data tracks for the *CCNK* promoter region, where the normalized count increase in the Br tumor relative to normal over the 10-kb region shown is 5.4-fold and for Co is 2.1-fold and the range for the other five tumors is 0.9-2.5. **d-e**) The two 1-Mb regions displayed in (c-d) were tiled with 1-kb bins and count density curves were fitted for all 7 tumor-normal pairs. Arrows mark the locations of indicated promoter peaks in the breast and colon tumors. **f**) Individual broad summits in (d-e) were zoomed-in and rescaled on *x*-axis centered over the indicated promoter peak and superimposed over raw normalized count tracks scaled to the height of the central peak.

A clue to the basis for the close clustering of the breast and colon tumor samples was revealed in the list of the top 100 hypertranscribed cCREs: Eighteen of the top 20 cCREs are located on Chromosome 17 (Figure S5a-f; Supplementary Table 1). Most of these cCREs are within either of two contiguous RNAPII-Ser5p-enriched regions (Chr17q12 and Chr17q21) of a few hundred kilobases in length in the breast tumor sample not seen in the adjacent normal tissue (Figure 7a-b). For the Co tumor sample a broad region of RNAPII enrichment is sharply defined within Chr17q21. The breadth of this region can explain why the Co sample was highly represented on Chromosome 17 based on the ranked cCRE list but showed no hypertranscription at the histone gene cluster. Major differences between the Br and Co tumors can be seen when sub-regions are group-autoscaled, which identified sharply defined promoter peaks just a few kilobases wide over RFFL, LIG3, ORMDL3 and CDK12 only in the Br tumor and MSL1 and ERBB2 in both the Br and Co tumor (Figure S5g-l).

To identify the likely source of regional hypertranscription, we searched PubMed for each of the 100 top-ranked genes with the word “cancer”. This revealed that the most frequently named gene in titles and abstracts by far is ERBB2 in Chr17q21 (35,121 PubMed hits), which accounts for 2/3 of the total, where the next most frequently named gene in the same Chr17q21 region is CDK12 (413 PubMed hits) (Supplementary Table 1). ERBB2 encodes Human Epidermal Growth Factor Receptor 2 (HER2), which is commonly amplified in breast and other tumors and is a target of therapy (26). As our measures of hypertranscription are scaled to the human genome sequence, amplification of a region will appear as a proportional increase in the level of RNAPII over the amplified region, so that we can interpret regional hypertranscription in both our Br and Co tumor samples as revealing independent amplification events.

To delineate possible RNAPII hypertranscription features within Chr17q12 and Chr17q21, we tiled 1-kb bins over each 1 Mb region centered on the highest peak in Chr17q21, corresponding to the the ERBB2 promoter in Chr17q21 and RFFL promoter in Chr17q12, and plotted count density within each bin with curve-fitting and smoothing. Remarkably, multiple broad summits appeared in both Br and Co tumor-versus-normal tracks (Figure 7d-e), and the six summits in the Br tumor sample accounted for the six highest ranked Chr17 promoter peaks (Figure S5a-f). We similarly plotted count densities of the four highest ranking cCREs outside of Chr17q12-21 (Supplementary Table 1), but tumor peaks in these regions were at least an order-of-magnitude lower than the ERBB2 peaks in the Br and Co tumor samples (Figure S6). Of the six summits in the BR sample ERBB2 and MSL1 also appeared in the Co sample, whereas no other tumor samples showed prominent summits above normal in Chr17q12-21 (Figure 7d-e). MSL1 encodes a subunit of a histone H4-lysine-16 acetyltransferase complex required for upregulation of the mammalian X chromosome (27).

We next superimposed each of the six summits in the Chr17q12-21 region in the Br tumor sample over the raw data tracks on expanded scales for clarity, centered over the highest promoter peak in the region (Figure 7f). For ERBB2, the ~100 kb broad summit is almost precisely centered over the ~1 kb wide ERBB2 promoter peak. Although the other summits are less broad, each is similarly centered over a promoter peak. Insofar as there are multiple summits much broader than the promoter peaks that they are centered over, our results are inconsistent with independent upregulation of promoters over the HER2 amplified regions. Rather, it appears that a HER2 amplification event was followed by clonal selection for broad regions around ERBB2 and other loci within each amplicon.

Interestingly, one of the summits in the Br sample absent from Co sample corresponds to the bidirectional promoters of MED1 and CDK12, both of which have been shown to functionally cooperate with co-amplified ERBB2 in aggressive breast cancer (28, 29). MED1 encodes a subunit of the 26-subunit Mediator complex, which regulates RNAPII pause release, and CDK12 is the catalytic subunit of the CDK12/Cyclin K kinase heterodimer complex, which phosphorylates RNAPII on Serine-2 for productive transcriptional elongation. We wondered whether the co-amplification of these RNAPII regulators might directly drive hypertranscription throughout the genome. As Cyclin K is the regulatory subunit of the CDK12 kinase, we would expect that the *CCNK* gene that encodes Cyclin K would be strongly upregulated in the Br tumor but not necessarily in the Co tumor. Indeed, we see a 5.4-fold increase in RNAPII-S5p over the *CCNK* promoter in the Br tumor relative to adjacent normal tissue, whereas in the Co tumor there is a 2.1-fold increase (Figure 7c), consistent with RNAPII hypertranscription directly driven in part by CDK1 amplification.

## Discussion

We have shown that hypertranscription at gene regulatory elements can be measured directly with FFPE-CUTAC. Whereas hypertranscription in cancer had been frequently documented in studies based on RNA-seq (2, 3), these indirect methods have limitations owing to variable processing of mRNAs, to the low level of mRNAs encoding critical regulatory proteins and to the need for accurate calibration to genomic DNA abundance. Crucially, none of the methods that have been applied to measure hypertranscription in cancer are suitable for FFPEs, which have long been standard for archival storage of tissue samples (12). Exposure of tissue to ~4% formaldehyde for days badly damages RNA and DNA and causes cross-links to form between tightly bound proteins and nucleic acids. However, this formaldehyde treatment also forms covalent bonds between DNA and lysine-rich histones in nucleosomes rendering them inflexible, so that open chromatin gaps are the only accessible DNA in the nucleus. By using antibodies to the phosphorylated RNAPII heptapeptide repeat present in 52 lysine-free tandem copies or to the abundant histone H3K27ac mark of active regulatory elements, FFPE-CUTAC takes advantage of the hyperaccessibility and abundance of the targeted epitope and the intractability of histone cross-linked chromatin to achieve exceptional signal-to-noise. As RNAPII FFPE-CUTAC maps the transcriptional machinery itself directly on the DNA regulatory elements, we obtain direct measurements of transcription initiation, as opposed to inferences based on estimating steady-state mRNA abundances. Thus, our mapping and quantitation of paused RNAPII, a critical checkpoint between transcriptional initiation and elongation, represents a powerful general approach to characterize hypertranscription at active regulatory elements genome-wide.

To quantify hypertranscription, we used normalized count differences between mouse tumor and normal tissue from the same FFPE sections and between matched human tumor and normal tissues, obviating the need for a spike-in normalization control. First, we mapped Tumor – Normal count differences for ENCODE-annotated cCREs, showing that nearly identical results were obtained regardless of whether the cCRE was a promoter, a proximal or distal enhancer or a CTCF (insulator) site. Second, we confirmed hypertranscription within these samples by examining replication-coupled histone clusters, which serve as proxies for cell proliferation. Third, we applied a completely unsupervised approach using our SEACR peak-caller to identify hypertranscribed loci throughout the genome. Remarkably, SEACR identified all of the 100 top-ranked of nearly 1 million human cCREs in at least one tumor (Supplementary Table 1), reporting a median of 3.7 overlapping cCREs in six of the seven different human tumors in our study. We also observed reductions in mitochondrial DNA that varied between tumors, suggestive of relaxed selection for mtDNA-encoded products during cancer progression. The rich regulatory information that can be extracted from RNAPII FFPE-CUTAC data using simple analytical tools, despite the use of sparse tissue samples in very poor condition relative to fresh or frozen samples, makes our method especially attractive for application of data mining tools that can be used to infer gene regulatory networks.

Finally, we observed that 55 of the overall top-ranked 100 human cCREs mapped to extensive regions of hypertranscription within Chromosome 17q12-21 in our Br and Co cancer samples, characteristic of HER2 amplifications, which are especially common in breast and colorectal cancer (30). HER2 amplifications are known to be subject to clonal selection, resulting in tumor heterogeneity (31), consistent with our observation of broad summits centered directly over promoters of candidate cancer driver genes within the amplified regions. Thus, three of the four loci showing apparent linkage disequilibrium around ERBB2 in our breast and colon tumor samples are known or potential cancer drivers, consistent with the observation of clonally heterogeneous HER2 amplifications in primary breast tumors by whole-genome sequencing (31). Clonal selection may be driven by selective sweeps (32) following amplification events that generate extrachromosomal DNA in double-minute acentric chromosomes which partition unequally during each cell division (33). Our evidence for linkage disequilibrium in the Br and Co samples that we analyzed suggests multiple selective sweeps resulting in loss of adjacent but physically unlinked DNA during evolution of these two tumors. Such copy number gains within a tumor can result in intra-tumor heterogeneity (17, 18) and are potential factors for resistance to therapy (34). FFPE-CUTAC thus potentially provides a general diagnostic strategy for detection and analysis of amplifications and clonal selection during cancer progression and therapeutic treatment.

Of the six broad summits observed in the breast tumor sample, those centered over ERBB2 and the bidirectional promoters of MED1 and CDK12 were already known to be associated with poor prognosis in HER2-positive breast cancer (28, 29), and MSL1 is part of the complex that upregulates the mammalian X-chromosome (27). Of particular interest is CDK12, a cyclin-dependent kinase that phosphorylates RNAPII on Serine-2 for pause release and transcriptional elongation and which is co-amplified with HER2 in ~90% of HER2^+^ breast cancers (35). We find that Cyclin K, the regulatory subunit of the CDK12/Cyclin K kinase complex is strongly upregulated in the same tumor, which suggests that amplification of CDK12 directly contributes to RNAPII hypertranscription and is in part responsible for poor prognosis in HER2/CDK12-amplified breast cancer patients (28, 35, 36). We envision the application of FFPE-CUTAC to cohorts of HER2-amplified and other cancer patient samples to ascertain the generality of our model for hypertranscription.

In summary, the high signal-to-noise and the abundance of RNAPII and H3K27ac epitopes used in FFPE-CUTAC have made possible detection of genome-wide hypertranscription using single 5 μm thick FFPE tissue sections ~1 cm^2^ in area and fewer than 4 million unique fragments. Our identification of HER2 amplifications and probable clonal selection events that did not rely on reference to any external data emphasizes the potential power of our approach for understanding basic genetic and epigenetic mechanisms underlying tumor evolution. The simple workflow of FFPE-CUTAC and its potential for scale-up and automation make it an attractive platform for retrospective studies and will require little modification for routine cancer screening and other personalized medicine applications.

## Methods

### Ethical statement

This research was approved by the Fred Hutch Institutional Animal Care and Use Committee (Protocol # 50842) and complies with all required ethical regulations.

### Mouse tumor and normal tissues and FFPEs

Ntva;cdkn2a−/− mice were injected intracranially with DF1 cells infected with and producing RCAS vectors encoding either PDGFB (21), ZFTA-RELA (19), or YAP1-FAM118b (20) as has been described (37). Upon weaning (~P21), mice were housed with same-sex littermates, with no more than 5 per cage and given access to food/water *ad libitum*. When the mice became lethargic and showed poor grooming, they were euthanized and their brains removed and fixed at least 48 hours in Neutral Buffered Formalin. All animal experiments were approved by and conducted in accordance with the Institutional Animal Care and Use Committee of Fred Hutchinson Cancer Center (Protocol #50842: Tva-derived transgenic mouse model for studying brain tumors). Tumorous and normal brains were sliced into five pieces and processed overnight in a tissue processor, mounted in a paraffin block and 10-micron sections were placed on slides. Mouse tissue (including normal and tumor bearing brains) were removed, fixed in 10% neutral-buffered formalin for a minimum of 24 hours and embedded into paraffin blocks. 10-μm serial sections were cut from formalin-fixed paraffin-embedded specimens and mounted on slides.

### Human FFPE slides

The following pairs of human tumor and adjacent normal 5 μm tissue sections from single FFPE blocks were purchased from Biochain, Inc: Breast Normal/Tumor cat. no. T8235086PP/PT; Colon Normal/Tumor cat. no. T8235090PP/PT; Kidney Normal/Tumor cat. no. T8235142PP/PT; Liver Normal/Tumor cat. no. T8235149PP/PT; Lung Normal/Tumor cat. no. T8235152PP/PT; Rectum Normal/Tumor cat. no. T8235206PP/PT; Stomach Normal/Tumor cat. no. T8235248PP/PT. Human primary liver tumor and normal samples were harvested from cases undergoing surgical resection at the University of Washington under the Institutional Review Board approved protocol and then subsequently deidentified.

### Antibodies

Primary antibodies: RNAPII-Ser5p: Cell Signaling Technologies cat. no. 13523, lot 3; RNAPII-Ser2p: Cell Signaling Technologies cat. no. 13499; H3K27ac: Abcam cat. no. ab4729, lot no. 1033973. Secondary antibody: Guinea pig α-rabbit antibody (Antibodies online cat. no. ABIN101961, lot 46671).

### On-slide FFPE-CUTAC

On-slide FFPE-CUTAC was performed as described (13) with modifications. Briefly, FFPE slides were placed in 800 mM Tris-HCl pH8.0 in a slide holder and incubated at 85-90°C for 1-14 hours, whereupon the paraffin melted and floated off the slide. Slides were cooled to room temperature and transferred to 20mM HEPES pH 7.5,150mM NaCl. Slides were drained and excess liquid wicked off using a Kimwipe tissue. The sections were immediately covered with 20-60 μL primary antibody in Triton-Wash buffer (20mM HEPES pH 7.5,150mM NaCl, 2mM spermidine and Roche complete EDTA-free protease inhibitor) added dropwise. Plastic film was laid on top to cover and slides were incubated ≥2 hr incubation at room temperature (or overnight at ~8°C) in a moist chamber. The plastic film was peeled back, and the slide was rinsed once or twice by pipetting 1 mL Triton-Wash buffer on the surface, draining at an angle. This incubation/wash cycle was repeated for the guinea pig anti-rabbit secondary antibody (Antibodies Online cat. no. ABIN101961) and for pAG-Tn5 preloaded with mosaic end adapters (Epicypher cat. no. 15-1117 1:20), followed by a Triton-Wash rinse and transfer of the slide to 10 mM TAPS pH 8.5. Tagmentation was performed in 5mM MgCl_2_, 10mM TAPS pH 8.5, 20% (v/v) N,N-dimethylformamide in a moist chamber and incubated at 55°C for 1 hr. Following tagmentation, slides were dipped in 10 mM TAPS pH 8.5, drained and excess liquid wicked off. Individual sections were covered with 2 μL 10% Thermolabile Proteinase K (TL ProtK) in 1% SDS using a pipette tip to loosen the tissue. Tissue was transferred to a thin-wall PCR tube containing 2 μL TL ProK using a watchmaker’s forceps, followed by 1 μL TL ProtK and transfer to the PCR tube. Tubes were incubated at 37°C for 30 min and 58°C for 30 min before PCR as described above.

### FFPE-CUTAC for curls

Curls were transferred to a 1.7 mL low-bind tube (Axygen cat. no. MCT-175-C), which tightly fits a blue pestle (Fisher cat. on. 12-141-364). Mineral oil (200 μl) was added and the tube was placed in a 85-90°C water bath for up to 5 min to melt the paraffin. The suspension was then homogenized ~10-20 sec with a pestle attached to a pestle motor (DWK Life Sciences cat no. 749540-0000). Warm cross-link reversal buffer (200 μl 800 mM Tris-HCl pH8.0) was added followed by addition of 6 μl of 1:10 Biomag amine paramagnetic beads (48 mg/ml, Polysciences cat. no. 86001-10). Homogenization was repeated, and 800 μl warm cross-link reversal buffer was added. Tubes were incubated at 85-90°C for 1-14 hours, vortexed, centrifuged briefly and the mineral oil was removed from the top without disturbing the surface. A 500 μl volume of mineral oil was added, mixed by inversion, centrifuged and the mineral oil removed leaving a thin oil layer. A 2.4 μl volume of agarose glutathione paramagnetic beads (Fisher cat. no. 88822) was added below the surface and mixed by inversion on a Rotator. Tubes were centrifuged briefly, placed on a strong magnet (Miltenyi Macsimag separator, cat. no. 130-092-168), and the supernatant removed and discarded, and the bead-bound homogenate was resuspended in up to 1 mL Triton-wash buffer (20 mM HEPES pH 7.5, 150 mM NaCl, 0.5 mM spermidine, 0.2mM EDTA, 0.05% Triton-X100 and Roche EDTA-free protease inhibitor) and divided into PCR tubes for antibody addition. Other steps through to library preparation and purification followed the standard FFPE-CUTAC protocol (13). Detailed step-by-step protocols for both slides and curls are available on Protocols.io: https://www.protocols.io/edit/cutac-for-ffpes-c5huy36w.

### DNA sequencing and data processing

The size distributions and molar concentration of libraries were determined using an Agilent 4200 TapeStation. Barcoded CUT&Tag libraries were pooled at equal volumes within groups or at approximately equimolar concentration for sequencing. Paired-end 50x50 bp sequencing on the Illumina NextSeq 2000 platform was performed by the Fred Hutchinson Cancer Research Center Genomics Shared Resources.

### Data analysis

#### Preparation of the CCREs

We obtained the mm10 and hg38 versions of the Candidate cis-Regulatory Elements by ENCODE (https://screen.encodeproject.org/) from UCSC (38). For mouse mm10 we used all 343,731 entries. Because our sequencing data was aligned to hg19, we used UCSC's liftOver tool to re-position the hg38 CCREs resulting in 924,834 entries. We noticed that many human CCREs were in repeated regions of the genome so we intersected the hg19 CCRE file with UCSC's RepeatMasked regions using bedtools 2.30.0 (39) "intersect -v" command to make a file of 464,749 CCREs not in repeated regions.

#### Preparation of histone regions

For mm10 we used these regions:

**Table:** 

chr13	21715711	21837530	H2bc13-H4bc2
chr13	22035122	22043658	H2ac12-H2bc11
chr13	23531044	23622558	H4c8-H1f4
chr13	23683473	23764412	H2ac6-H1f1

For hg19 we used these regions:

**Table:** 

chr1	149783434	149859466	Minor
chr6	26017260	26285727	Major

#### Alignment of PE50 Illumina sequencing

1. We used cutadapt 2.9 (40) with parameters "-j 8 --nextseq-trim 20 -m 20 -a AGATCGGAAGAGCACACGTCTGAACTCCAGTCA -AAGATCGGAAGAGCGTCGTGTAGGGAAAGAGTGT -Z" to trim adapters from 50bp paired-end reads fastq files.

2. We used Bowtie2 2.4.4 (41) with options "--very-sensitive-local --soft-clipped-unmapped-tlen --dovetail --no-mixed --no-discordant -q --phred33 -I 10 -X 1000" to map the paired-end 50bp reads to the mm10 Mus musculus or hg19 Homo sapiens reference sequences obtained from UCSC.

3. We used samtools 1.14 (42) "view" to extract properly paired reads from the mm10 alignments into bed files of mapped fragments.

4. We computed the fraction of fragments mapped to chrM.

5. We used bedtools 2.30.0 "genomecov" to make a normalized count track which is the fraction of counts at each base pair scaled by the size of the reference sequence so that if the counts were uniformly distributed across the reference sequence there would be one at each position.

6. We ran Picard 2.18.29 MarkDuplicates program (http://broadinstitute.github.io/picard/) on the sam output from bowtie2.

#### Preparation of aligned samples

1. For mouse, we used all mapped fragments. For human, we removed duplicates as marked by Picard from the sam files before making normalized count tracks.

2. For mouse on-slide experiments, we merged tumor replicates within the experiment. For human, we merged mapped fragments from 5 different experiments for each tumor and then equalized the numbers of fragments for tumor and normal pairs by downsampling the larger of the two using the UNIX shuf command.

#### Peak-finding

We ran SEACR 1.3 (25) with parameters "norm relaxed" on tumor samples with the normal sample from each tumor and normal pair as the control. For comparison, we also called peaks after reversing the roles of tumor and normal.

#### Preparation of the per-cCRE and per-Histone region files

We used the bedtools intersect and groupby commands to sum the number of normalized counts from the tracks within the cCRE and histone region boundaries. Because the cCREs and histone regions vary in size, we then averaged the number of normalized counts within each to make them more comparable. The resulting files have one row per cCRE or histone region and one column per sample and are suitable for submission to the Degust server (https://degust.erc.monash.edu/) using the Voom/Limma option (−log_10_FDR versus log_2_FoldChange).

#### Preparation of Tumor-Normal files

We computed Tumor-Normal pairs from the CCRE region files and sorted them by largest differences in absolute value (Supplementary Table 1).

#### Curve-fitting

We partitioned the genome into 1 kb tiles and merged replicates, then downsampled to equalize library sizes between tumor and normal samples from each patient and added up normalized counts within each tile. For each tumor and normal patient sample, we fit the normalized counts across tiles using a Local Polynomial Regression (LOESS) model as implemented in the ᠘stats᠙ package of the R programming language, setting the degree of smoothing to 0.2 (Figure 7c-d) or 0.5 (Figure 7e) specified by the ᠙span᠙ parameter of ᠙loess᠙ function.

#### UMAPs

To ensure the quality of samples for downstream analysis, we excluded samples with fewer than 100,000 read counts or less than 10,000,000 bp of total fragment length. We utilized cCRE regions as the genomic features and calculated the raw sequencing read count overlapping each cCRE region using the "getCounts" function from the chromVAR R package. Processing the cCRE regions by samples count matrix, we initially applied the term frequency-inverse document frequency (TF-IDF) normalization method (43). This method first normalizes read counts across samples to correct for differences in total read depth and then adjusts across cCRE regions, assigning higher values to rarer regions. TF-IDF normalization was implemented using the "RunTFIDF" function from the Signac R package. This step was followed by the selection of top features using "FindTopFeatures" from the Signac package and data scaling performed by "ScaleData" from the Seurat package. Subsequently, we conducted principal component analysis (PCA) on the scaled data and then used the top 50 principal components to generate a UMAP representation, providing a refined visualization of the relationship across samples.

### Statistics and Reproducibility

No statistical method was used to predetermine sample size nor were data were excluded from the analyses. The experiments were not randomized and Investigators were not blinded to allocation during experiments and outcome assessment.

## Supplementary Material

Supplement 1

Supplement 2

## Data Availability

The sequencing data generated in this study have been deposited in the NCBI GEO database under accession code GSE261351.

## Code Availability

Custom scripts used in this study are available from GitHub: https://github.com/Henikoff/FFPE.
