# Supplementary material for: RNA Polymerase II hypertranscription in cancer FFPE samples
Source: bioRxiv. 2024 May 21:2024.02.28.582647. Preprint. [Version 3] doi: 10.1101/2024.02.28.582647 (PMC10979862; doi:10.1101/2024.02.28.582647)
Supplement: Supplement 2 [file NIHPP2024.02.28.582647v3-supplement-2.pdf]

bioRxiv preprint doi: https://doi.org/10.1101/2024.02.28.582647; this version posted May 21, 2024. The copyright holder for this preprint (which was not certified by peer review) is the author/funder, who has granted bioRxiv a license to display the preprint in perpetuity. It is made available under aCC-BY 4.0 International license.

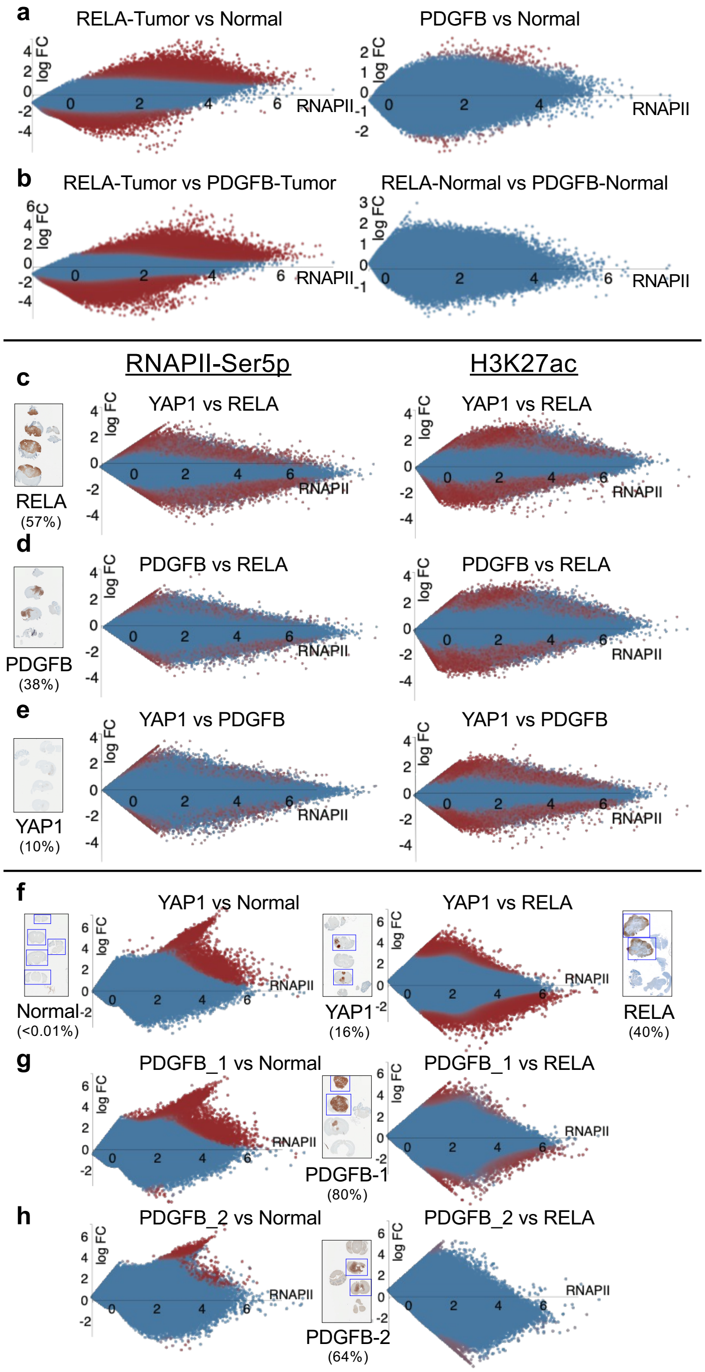

Figure S1 | RNAPII-Ser5p FFPE-CUTAC shows stronger and more frequent changes in up-regulation than down-regulation of cCREs. Related to Figure 1. The Voom/Limma option of the Degust server (https://degust.erc.monash.edu/) was applied to mouse cCRE RNAPII-Ser5p FFPE-CUTAC data from pooled replicates from 5 RELA and 4 PDGFB experiments. MA plots display $x = \log_{10}(\text{Tumor*Normal})/2$ versus $y = \log_2(\text{Tumor/Normal})$ for normalized counts from the tumor and normal samples being compared, and red color indicates FDR < 0.05. Normalized counts are the fraction of counts at each base pair scaled by the size of the Mm10 reference sequence (2,818,974,548), so that if the counts are uniformly distributed across the reference sequence there would be one at each position. (**a-b**) Both RELA and PDGFB tumor sections show higher counts than normal sections but significant RELA changes both up and down are far stronger than PDGFB changes, confirmed in a head-to-head comparison between tumors and normal sections. (**c-e**) Same as (a-b) except using either RNAPII-Ser5p or histone H3K27ac antibodies for FFPE-CUTAC and using entire 10 µm curls divided into 4-8 samples per curl for PCR and sequencing. For MA plots, data were merged from multiple experiments and equalized by downsampling to 10 million fragments, with 4 merged replicates per sample. DAP-stained slides for each paraffin block used, with the total fraction of tumor indicated in parentheses. (**f-h**) Voom/Limma was used to construct MA plots based on individual 10 µm sections from single slides corresponding to the boxed sections on slides DAP-stained for tumor-driver transgene expression. Numbers in parentheses are percentages of tumor cells based on numbers of stained and unstained cells within the boxed sections.

bioRxiv preprint doi: https://doi.org/10.1101/2024.02.28.582647; this version posted May 21, 2024. The copyright holder for this preprint (which was not certified by peer review) is the author/funder, who has granted bioRxiv a license to display the preprint in perpetuity. It is made available under aCC-BY 4.0 International license.

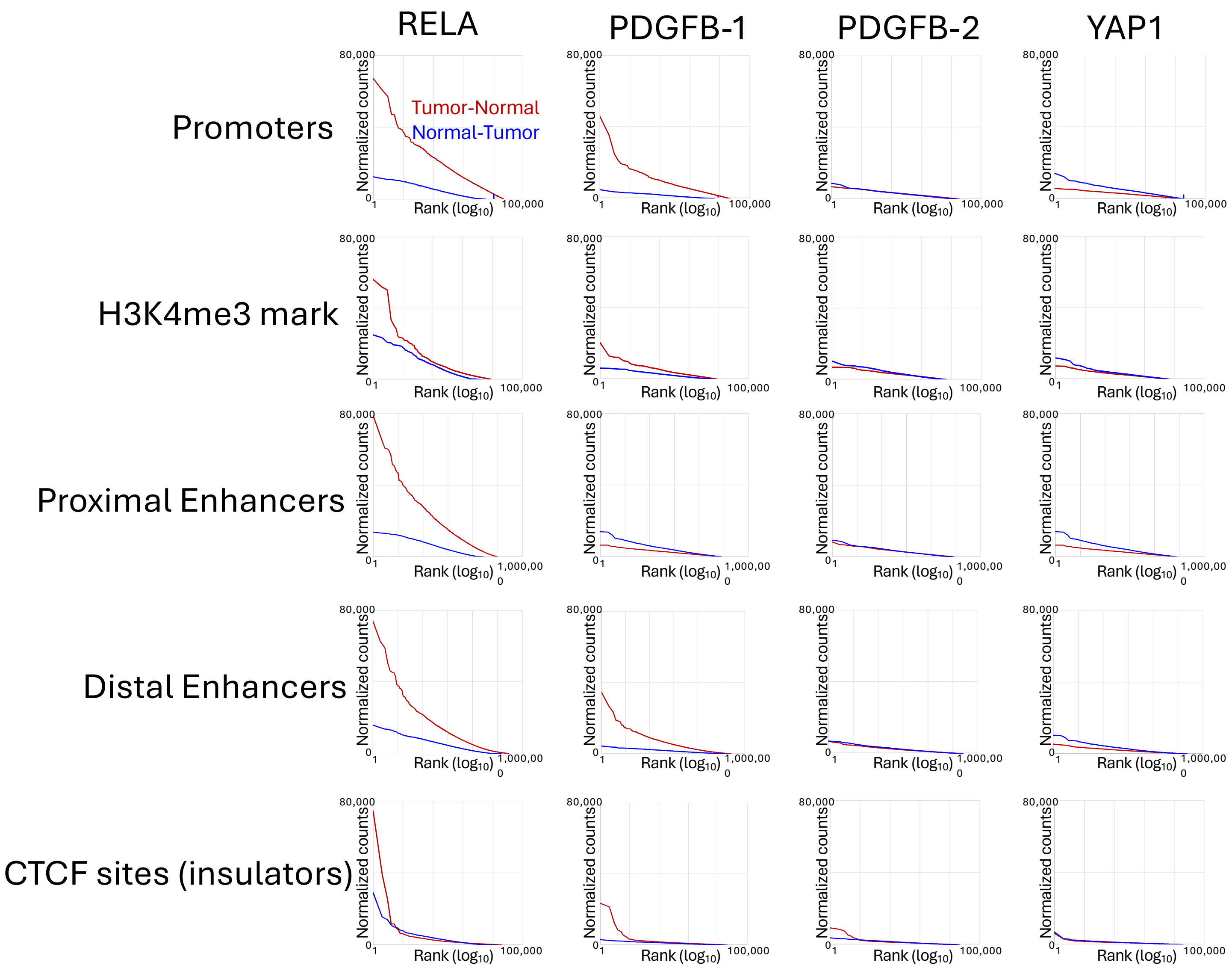

Figure S2 | Hypertranscription mapped over the 343,731 ENCODE-annotated mouse cCREs categorized by regulatory element type. Related to Figure 1. For each tumor and normal sample, we counted the number of mapped fragments spanning each base-pair in a cCRE scaled to the mouse genome and averaged the number of counts over that cCRE. We then divided up the 343,731 cCREs into the five ENCODE-annotated categories: Promoters (24,114), H3K4me3-marked cCREs (10,538), Proximal Enhancers (108,474), Distal Enhancers (211,185) and CTCF cCREs (24,072) and rank-ordered based on tumor minus normal representing global upregulation, and conversely rank-ordered cCREs based on normal minus tumor representing global downregulation. With such a large collection of loci, our a priori expectation is that the rank-ordered distribution of differences between tumor and normal will be approximately the same regardless of whether the differences are based on tumor minus normal or normal minus tumor. For clarity, we plotted rank-ordered differences on a log10 scale. Strong hypertranscription for RELA and PDGFB-1, weak hypertranscription for PDGFB-2 and little or no hypotranscription for YAP1 is seen for all classes, consistent with the Bland-Altman plots shown in Figure 1b-e.

bioRxiv preprint doi: https://doi.org/10.1101/2024.02.28.582647; this version posted May 21, 2024. The copyright holder for this preprint (which was not certified by peer review) is the author/funder, who has granted bioRxiv a license to display the preprint in perpetuity. It is made available under aCC-BY 4.0 International license.

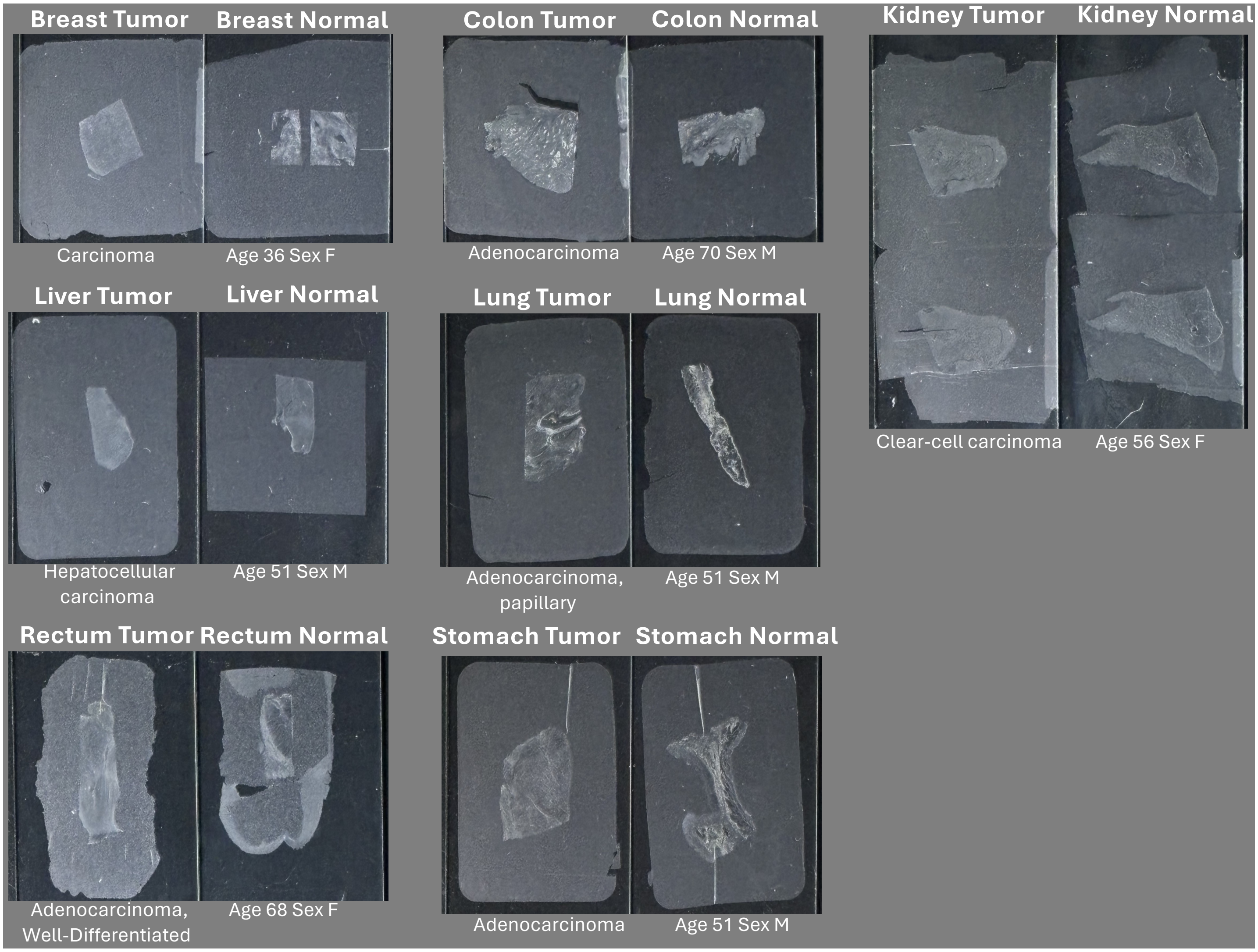

Figure S3 | Photographs of 5 µm FFPE sections from human tumor and adjacent normal tissues. Related to Figure 2. Pathology classification, age and sex were provided by the vendor (BioChain). Each image spans the width of a standard charged microscope slide, where the tissue is visible under the paraffin skin. On-slide RNAPII-Ser5p FFPE-CUTAC was applied to slides in parallel, using a total of four slides each for 100 separate samples in all to produce the data analyzed in this study. To avoid the impression that these individual tumors are representative of their tumor types, we abbreviate their designations: Br, Co, Ki, Li, Lu, Re and St.

bioRxiv preprint doi: https://doi.org/10.1101/2024.02.28.582647; this version posted May 21, 2024. The copyright holder for this preprint (which was not certified by peer review) is the author/funder, who has granted bioRxiv a license to display the preprint in perpetuity. It is made available under aCC-BY 4.0 International license.

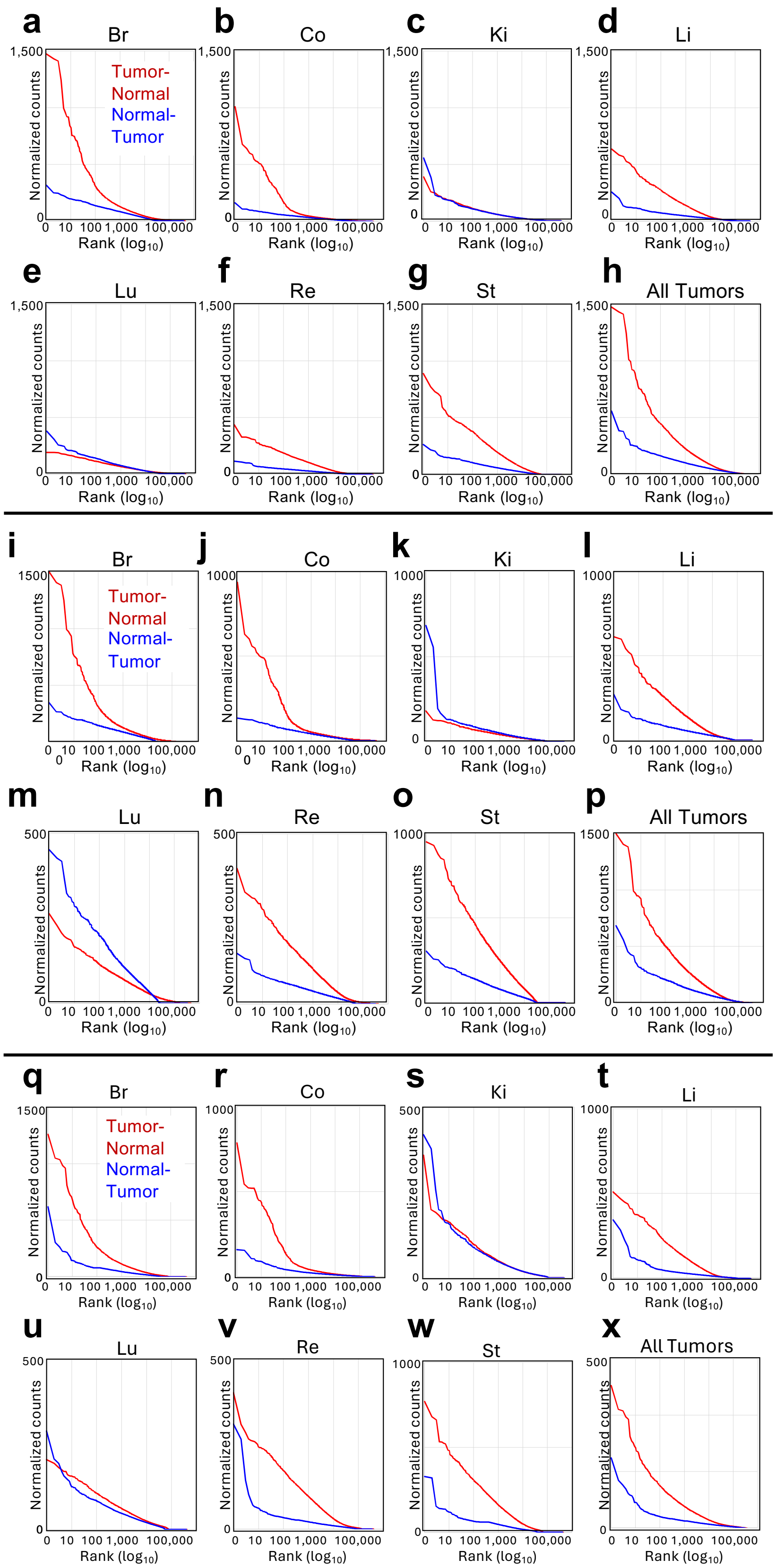

Figure S4 | Hypertranscription in human Tumor-vs-Normal tissues. Related to Figure 2. a-h) Same data as in Figure 2a-h, except plotted as in Figure S3 to facilitate comparisons. **i-p**) Combined data from a single slide with duplicate removal. **q-x)** Combined data from 4 slides after removing duplicates and equalizing the number of fragments between tumor and normal sections. Number of unique fragments per sample in each Tumor/Normal pair: Br: 1,125,608; Co: 3,712,097; Ki: 2,031,893; Li: 2,983,411; Lu: 1,123,638; Re: 3,284,736; St: 719,598.

bioRxiv preprint doi: https://doi.org/10.1101/2024.02.28.582647; this version posted May 21, 2024. The copyright holder for this preprint (which was not certified by peer review) is the author/funder, who has granted bioRxiv a license to display the preprint in perpetuity. It is made available under aCC-BY 4.0 International license.

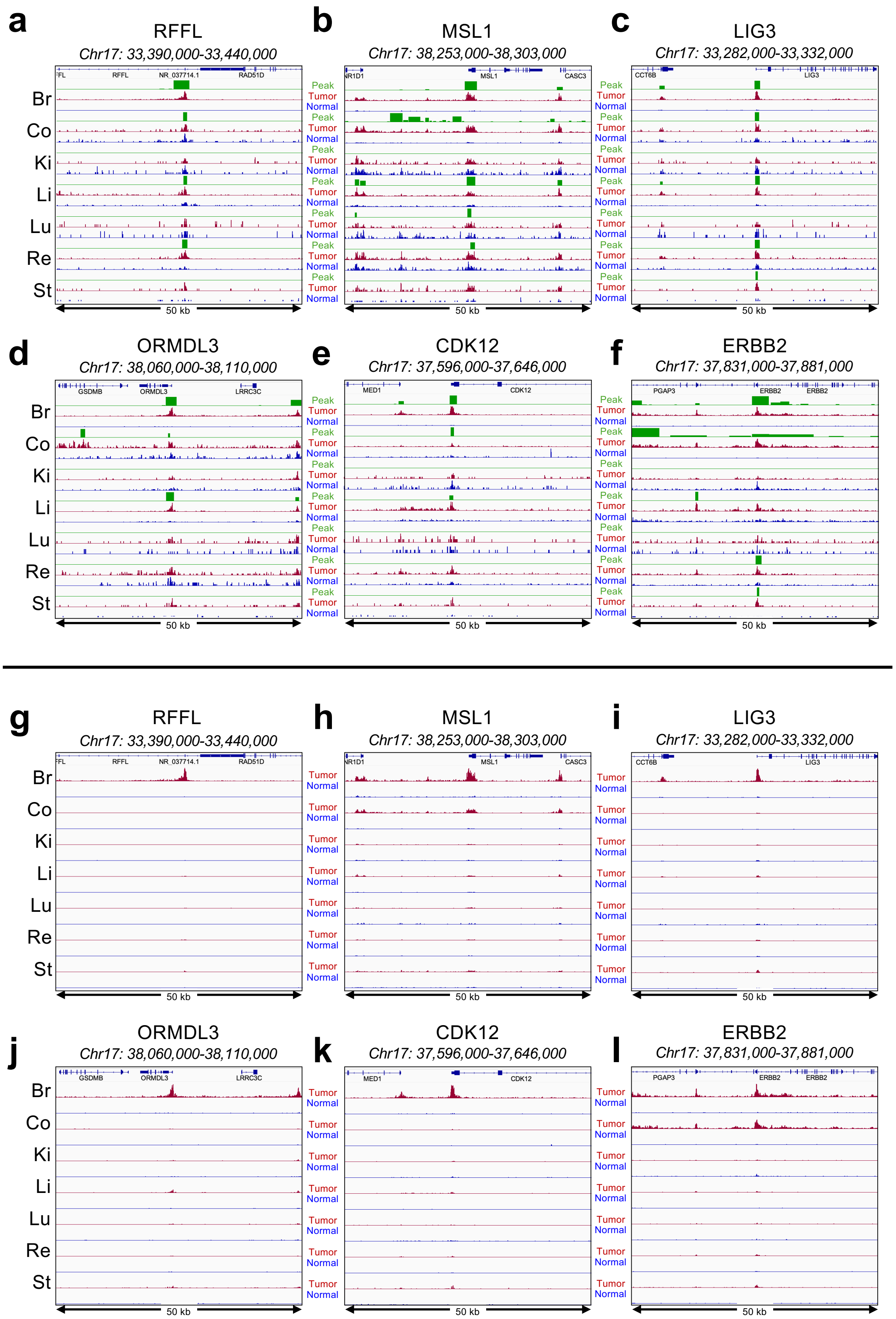

Figure S5 | Focal hypertranscribed regulatory elements embedded in broad regions of hypertranscription on Chromosome 17q12-22. Related to Figure 7. a-f) The six most highly transcribed cCREs within the ~5 Mb region of Chromosome 17q1.2-2.2 are displayed with each tumor (dark red) and normal (blue) pair scaled to one another so that peaks can be observed in all samples. SEACR peaks (green) are group-autoscaled in all panels. **g-l**) Same as (a-f) except that all tumor-normal samples are group-autoscaled to the height of the tallest peak, where the disappearance of all the peaks except for those in Br and for MSL1 and ERBB2 in Co is evidence that peaks in these regions are strongly hypertranscribed in Br and partially in Co but not in any of the other tumors.

bioRxiv preprint doi: https://doi.org/10.1101/2024.02.28.582647; this version posted May 21, 2024. The copyright holder for this preprint (which was not certified by peer review) is the author/funder, who has granted bioRxiv a license to display the preprint in perpetuity. It is made available under aCC-BY 4.0 International license.

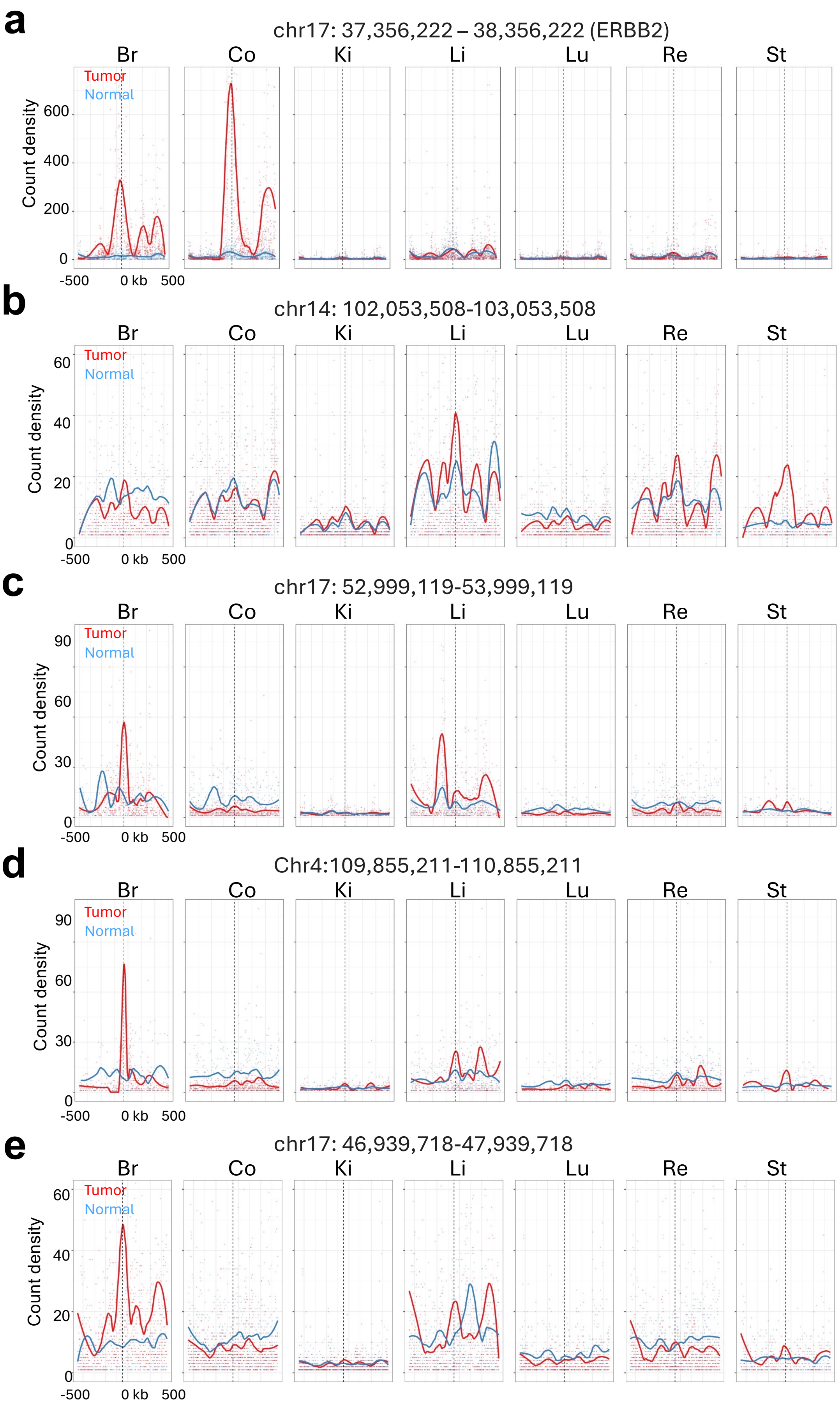

Figure S6 | Weak RNAPII upregulation of RNAPII of the top-ranked loci outside of the HER2 amplicon. Related to Figure 7. See Figure 7c-d for details.
