# Supplementary material for: Direct measurement of RNA Polymerase II hypertranscription in cancer FFPE samples
Source: bioRxiv. 2024 Mar 16:2024.02.28.582647. Preprint. [Version 2] doi: 10.1101/2024.02.28.582647 (PMC10979862; doi:10.1101/2024.02.28.582647)
Supplement: Supplementary file 1 [file NIHPP2024.02.28.582647V2-supplement-1.pdf]

bioRxiv preprint doi: https://doi.org/10.1101/2024.02.28.582647; this version posted March 16, 2024. The copyright holder for this preprint (which was not certified by peer review) is the author/funder, who has granted bioRxiv a license to display the preprint in perpetuity. It is made available under aCC-BY 4.0 International license.

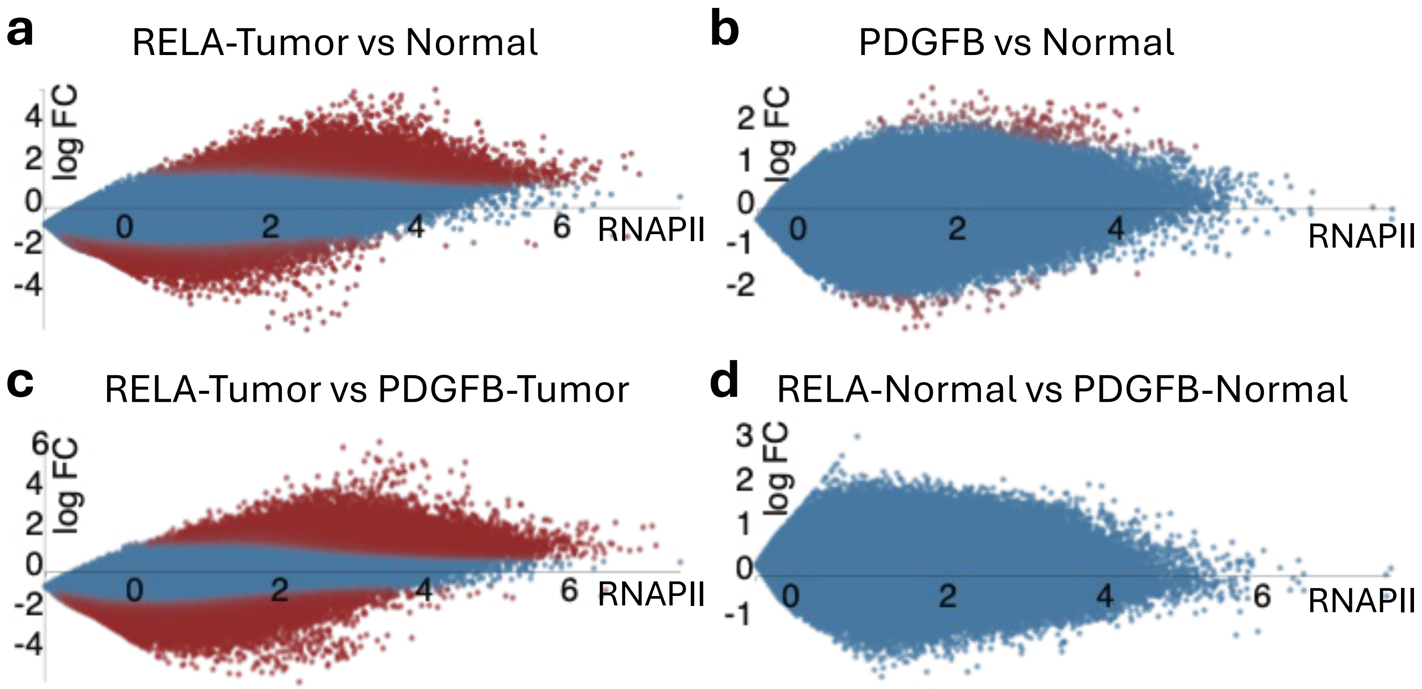

Supplementary Figure 1 | RNAPII-Ser5p FFPE-CUTAC shows stronger and more frequent changes in up-regulation than down-regulation of cCREs. The Voom/Limma option of the Degust server (https://degust.erc.monash.edu/) was applied to mouse cCRE RNAPII-Ser5p FFPE-CUTAC data from pooled replicates from 5 RELA and 4 PDGFB experiments. MA plots display $x = \log_2(\text{Tumor*Normal})/2$ (average log RNAPII) and $y = \log_2(\text{Tumor/Normal})$ (log fold-change) for normalized counts from samples (Tumor and Normal) being compared, and red color indicates indicates FDR < 0.05. Normalized counts are the fraction of counts at each base pair scaled by the size of the Mm10 reference sequence (2,818,974,548), so that if the counts are uniformly distributed across the reference sequence there would be one at each position. Both (**a**) RELA and (**b**) PDGFB tumor sections show higher counts than normal sections but significant RELA changes both up and down are far stronger than PDGFB changes, confirmed in a head-to-head comparison between (**c**) tumors and (**d**) normal sections.

bioRxiv preprint doi: https://doi.org/10.1101/2024.02.28.582647; this version posted March 16, 2024. The copyright holder for this preprint (which was not certified by peer review) is the author/funder, who has granted bioRxiv a license to display the preprint in perpetuity. It is made available under aCC-BY 4.0 International license.

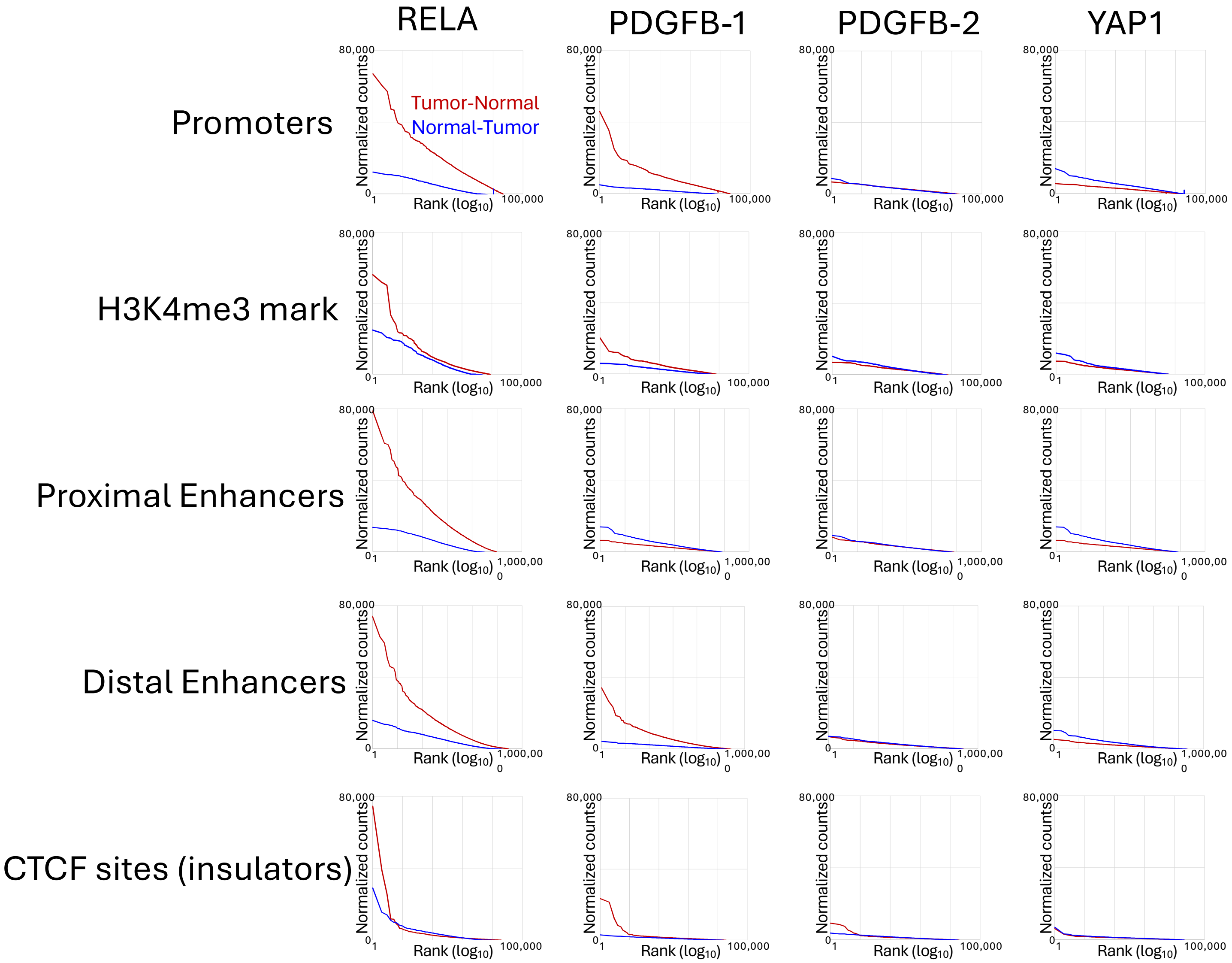

Supplementary Figure 2 | Hypertranscription mapped over the 343,731 ENCODE-annotated mouse cCREs categorized by regulatory element type. See Figure 3b-e for details.

bioRxiv preprint doi: https://doi.org/10.1101/2024.02.28.582647; this version posted March 16, 2024. The copyright holder for this preprint (which was not certified by peer review) is the author/funder, who has granted bioRxiv a license to display the preprint in perpetuity. It is made available under aCC-BY 4.0 International license.

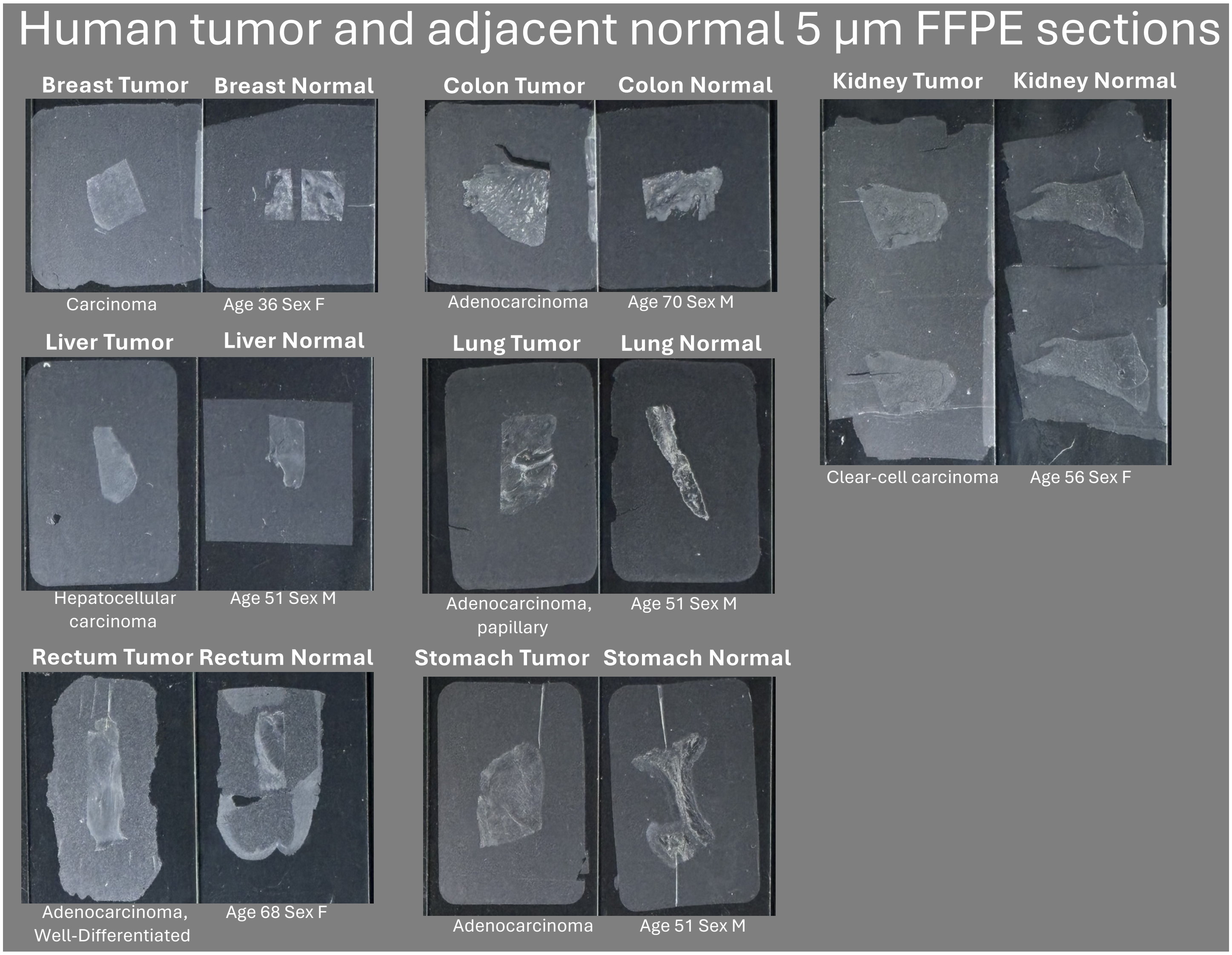

Supplementary Figure 3 | Photographs of 5 µm FFPE sections from human tumor and adjacent normal tissues. Pathology classification, age and sex were provided by the vendor (BioChain). Each image spans the width of a standard charged microscope slide, where the tissue is visible under the paraffin skin. On-slide RNAPII-Ser5p FFPE-CUTAC was applied to slides in parallel, using a total of four slides each for 100 separate samples in all to produce the data analyzed in this study.

bioRxiv preprint doi: https://doi.org/10.1101/2024.02.28.582647; this version posted March 16, 2024. The copyright holder for this preprint (which was not certified by peer review) is the author/funder, who has granted bioRxiv a license to display the preprint in perpetuity. It is made available under aCC-BY 4.0 International license.

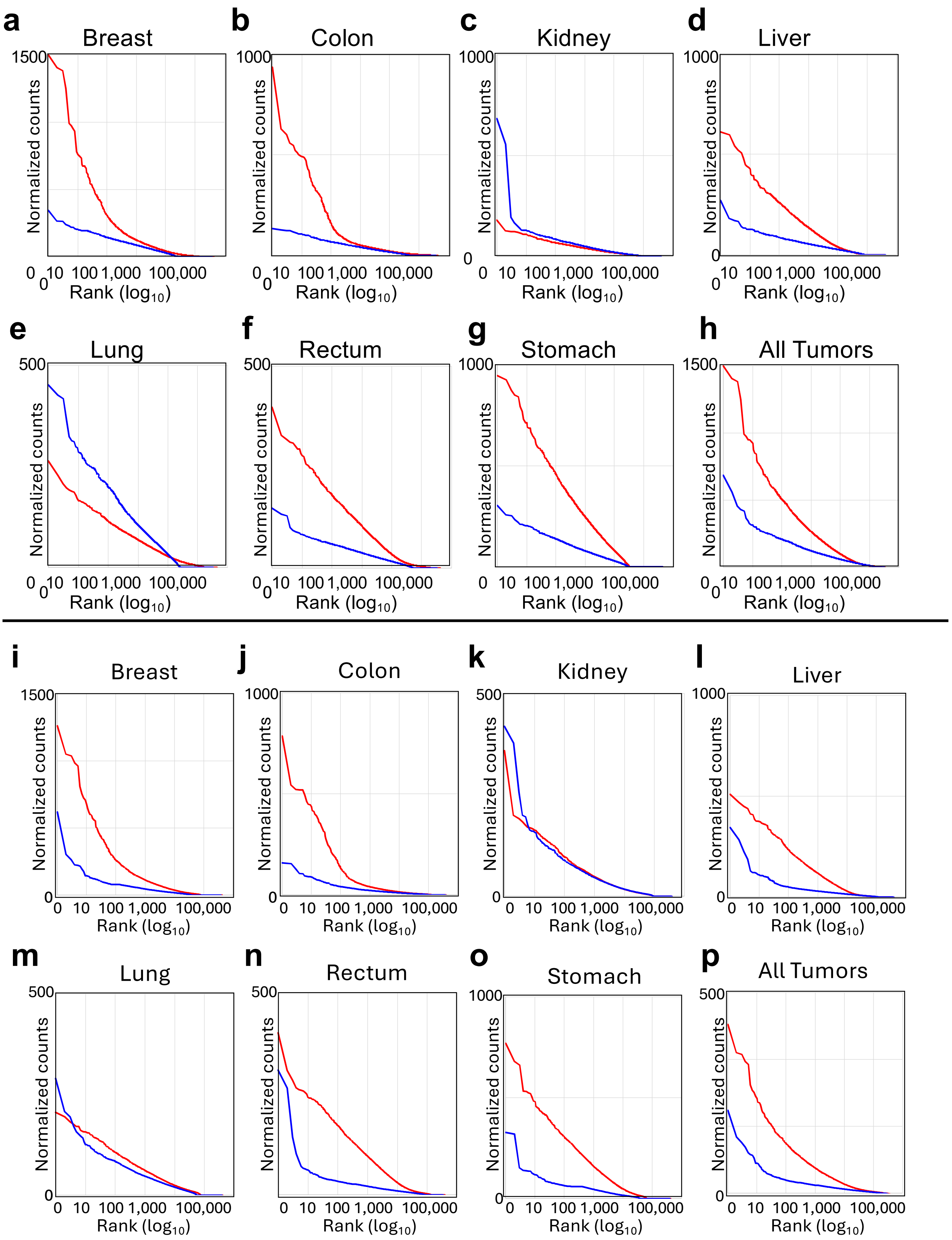

Supplementary Figure 4 | Hypertranscription in human Tumor-vs-Normal tissues: **a-h**) Combined data from a single slide with duplicate removal. **i-p)** Combined data from 4 slides after removing duplicates and equalizing the number of fragments between tumor and normal sections. Number of fragments per sample in each Tumor/Normal pair: Breast: 1,125,608; Colon: 3,712,097; Kidney: 2,031,893; Liver: 2,983,411; Lung: 1,123,638; Rectum: 3,284,736; Stomach: 719,598.

bioRxiv preprint doi: https://doi.org/10.1101/2024.02.28.582647; this version posted March 16, 2024. The copyright holder for this preprint (which was not certified by peer review) is the author/funder, who has granted bioRxiv a license to display the preprint in perpetuity. It is made available under aCC-BY 4.0 International license.

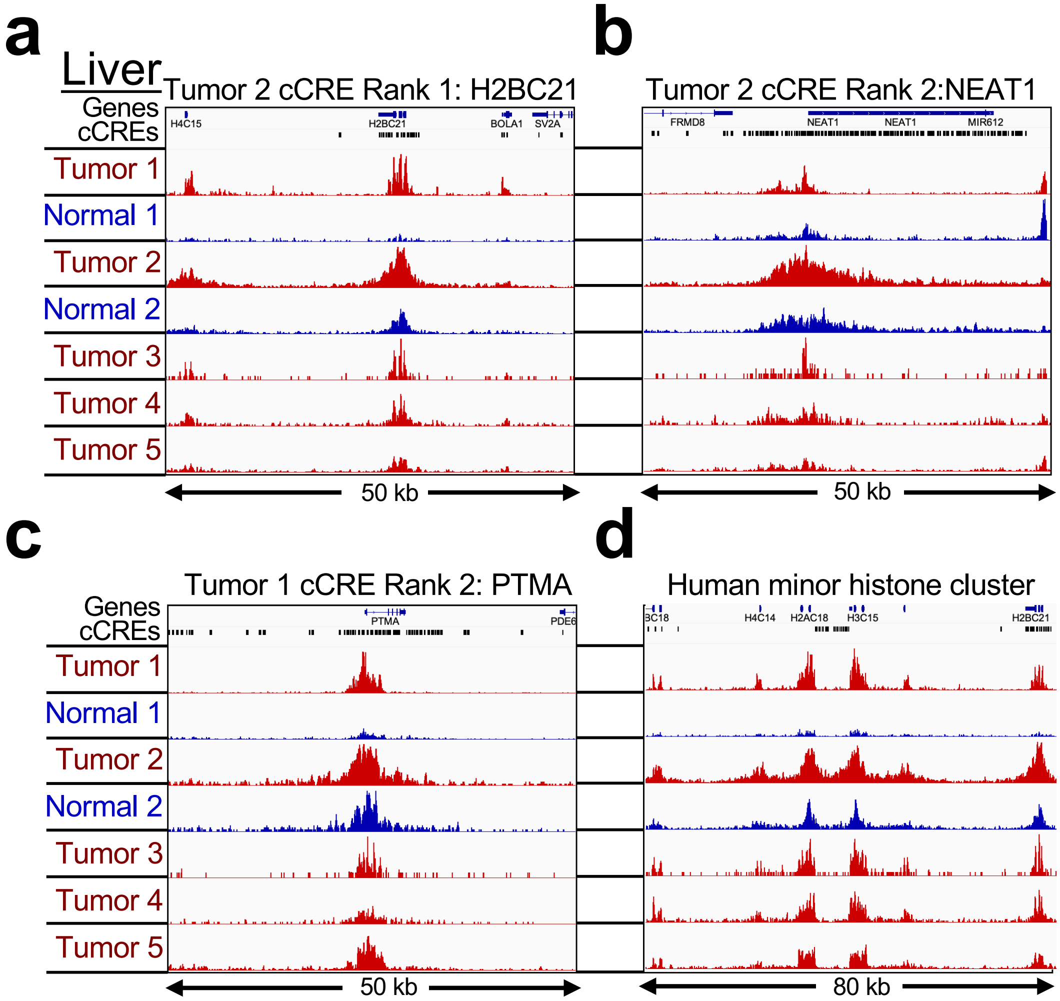

Supplementary Figure 5 | Top-ranked human cCREs based on hypertranscription correspond to SEACR Tumor-vs-Normal RNAPII-Ser5p peaks. a-d) Comparison of tracks for high-ranking cCREs. See Figure 7a-d for details. **e**) Same as (a) for the minor histone cluster. **f**) Tracks for the 500-kb region on Chromosome 17q1.2 with the most high-ranking cCREs for both the Breast and Colon samples reveal broad regions of prominent hypertranscription, indicative of likely HER2 amplifications in both tumors.

bioRxiv preprint doi: https://doi.org/10.1101/2024.02.28.582647; this version posted March 16, 2024. The copyright holder for this preprint (which was not certified by peer review) is the author/funder, who has granted bioRxiv a license to display the preprint in perpetuity. It is made available under aCC-BY 4.0 International license.

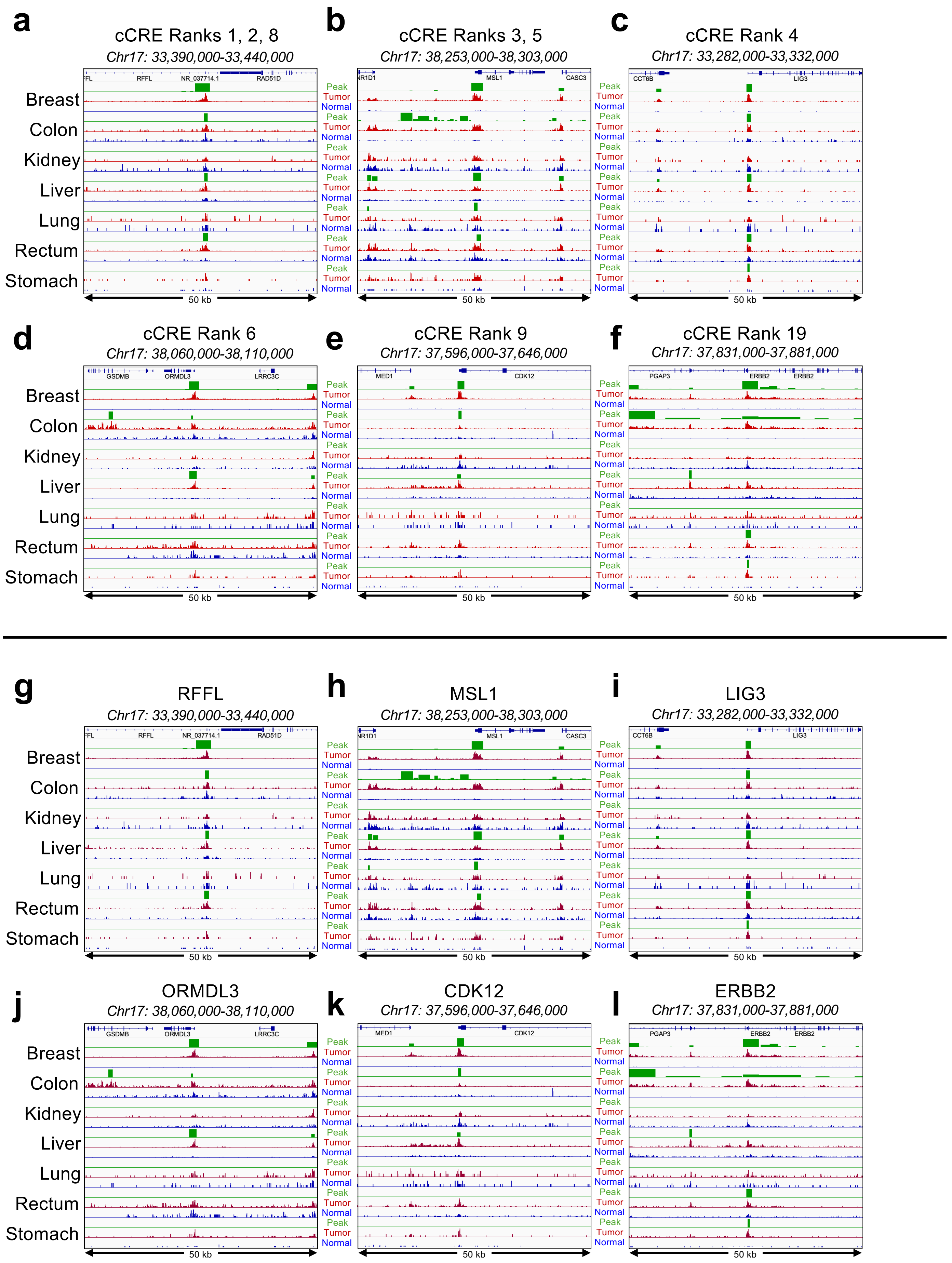

Supplementary Figure 6 | Hypertranscription differs between human liver tumors. a-d) Top-ranked cCREs based on liver tumors 1 and 2 (red) and matched normal (blue) counts. Tumor/Normal tracks and Tumors 3-5 are group-autoscaled.
